# Required minimal protein domain of flower for synaptobrevin2 endocytosis in cytotoxic T cells

**DOI:** 10.1007/s00018-024-05528-1

**Published:** 2024-12-18

**Authors:** Keerthana Ravichandran, Claudia Schirra, Katja Urbansky, Szu-Min Tu, Nadia Alawar, Stefanie Mannebach, Elmar Krause, David Stevens, C. Roy D. Lancaster, Veit Flockerzi, Jens Rettig, Hsin-Fang Chang, Ute Becherer

**Affiliations:** 1 Cellular Neurophysiology, Center for Integrative Physiology and Molecular Medicine (CIPMM), Saarland University, 66421 Homburg, Germany; 2 Department of Structural Biology, Center of Human and Molecular Biology (ZHMB), Faculty of Medicine Building 60, Saarland University, 66421 Homburg, Germany; 3 Experimental and Clinical Pharmacology and Toxicology and Preclinical Center for Molecular Signaling, Saarland University, Homburg, Germany

**Keywords:** CACFD1, Endocytosis, Lytic granule, CD8 + lymphocyte, Immunological synapse

## Abstract

Flower, a highly conserved protein, crucial for endocytosis and cellular fitness, has been implicated in cytotoxic T lymphocyte (CTL) killing efficiency through its role in cytotoxic granule (CG) endocytosis at the immune synapse (IS). This study explores the molecular cues that govern Flower-mediated CG endocytosis by analyzing uptake of Synaptobrevin2, a protein specific to CG in mouse CTL. Using immunogold electron microscopy and total internal fluorescence microscopy, we found that Flower translocates in a stimulus-dependent manner from small vesicles to the IS, thereby ensuring specificity in CG membrane protein recycling. Using confocal live-cell imaging, we assessed the ability of a range of naturally occurring mouse, human and *Drosophila* isoforms to rescue defective endocytosis in Flower KO CTLs. This analysis demonstrated that the N-terminal portion of the protein, encompassing amino acids 1–106 in mice, is the minimal domain necessary for Synaptobrevin2 endocytosis. Additionally, we identified two pivotal sites through site-specific mutation: a putative AP2-binding site, and a tyrosine at position 104 in mouse Flower. These findings provide insights into Flower's specific functional domain essential for CG endocytosis, which is a key process in mediating T cell serial killing required for the effective fight against cancer.

**Supplementary Information:**

The online version contains supplementary material available at 10.1007/s00018-024-05528-1.

## Introduction

The protein Flower (Fwe; CACFD1) was discovered in a genetic screen for mutations affecting synaptic transmission in *Drosophila* [1]. It is an integral membrane protein present on vesicles and at the periactive zone of the synapse. Fwe is required for maintaining synaptic function at the *Drosophila* neuromuscular junction via the promotion of endocytosis [1]. Similarly, Fwe has been shown to promote endocytosis in cytotoxic T lymphocytes (CTLs) [2]. This protein has been identified with alternative splice variants in various organisms. *Drosophila* expresses three isoforms (dUbi, dLoseA, dLoseB), whilst mice (mFwe1-4) and humans (hFwe1-4) express four [1, 3, 4]. In addition to its role in endocytosis, Fwe is also involved in cell competition, where the different isoforms either support the survival of cells (winner isoform) or not (loser isoform) [3, 5, 6]. It remains to be shown whether the different isoforms also have different functions in endocytosis and whether there is a relation to cell competition.

CTLs are an ideal cell type for investigating Fwe function because they are highly dependent on endocytosis for serial killing, which is required for the effective function of the immune response. CTLs are the effector cells of the adaptive immune system [7], recognizing and eliminating virus-infected and cancer cells. Upon recognition of a target cell, the CTL develops an area of close contact, referred to as the immune synapse (IS) [8, 9]. Cytotoxic granules (CGs) are delivered to the IS and fuse with the plasma membrane via the SNARE complex, ultimately releasing cytotoxic proteins such as perforin and granzymes [10]. Synaptobrevin2 (Syb2) is the v-SNARE of the CG SNARE complex in mouse cells [11]. CTLs contain low numbers of CGs [12] and rely on the recycling of CG components such as Syb2 via clathrin-mediated endocytosis for efficient serial killing [13]. The loss of Fwe reduces the killing efficacy of CTL without directly affecting the fusion of CG. A degranulation assay using LAMP1 as CG membrane marker was not affected by Fwe knockout (KO) CTLs [2]. In contrast, Fwe deletion resulted in a strong reduction in the endocytosis of Syb2 in CTL. The expression of full-length mouse Fwe (mFwe2) rescued Syb2 endocytosis in Fwe KO CTLs, demonstrating that the deficit in endocytosis is a result of the lack of Fwe [2]. Fwe contains two YXXΦ motifs, which are putative AP2 binding sites [14] and may function in interactions with clathrin during endocytosis. Deletion of both sites renders Fwe unable to rescue endocytosis in mouse Fwe KO CTLs [2, 15]. We next asked whether Fwe is a general player in endocytosis of CG membrane proteins, where it is localized and which domains are required to fulfill its function. Measurement of transferrin receptor 1 (TfR1) internalization via FACS revealed that Fwe is not generally required for clathrin-mediated endocytosis. Instead, we showed that it specifically promotes the uptake of CG membrane proteins Syb2 and LAMP1. Using total internal fluorescence microscopy (TIRFM) and immunogold electron microscopy, we found that Fwe is transported to the plasma membrane following IS formation. These results suggest that Fwe serves as a platform for CG membrane protein endocytosis. We then studied the ability of the eleven known Fwe splice variants to rescue endocytosis of Syb2 in mouse CTLs lacking Fwe. Our findings demonstrate that the C-terminal end of the protein is dispensable. Additional targeted mutations indicate that the AP2 interaction site at the N-terminus as well as a highly conserved tyrosine is required for Fwe function. We thereby identified the minimal protein domain of Fwe responsible for Syb2 endocytosis.

## Materials and methods

### Mice

Fwe knock-out mice (Fwe KO) were generated as described previously [2]. Fwe-KO mice and WT mice (Charles River) used in this study were all C57BL/6N background. Animals used for experiments were 15–22 weeks old and of either sex. Animals were kept at 22 °C room temperature with 50–60% humidity and 12 h dark/light cycles. All experimental procedures were approved and performed according to the regulations by the state of Saarland (Landesamt für Verbraucherschutz, AZ.: 2.4.1.1).

### Cell culture

Splenocytes were isolated from C57BL/6N WT and Fwe KO mice as described [34]. Briefly, naive CD8^+^ T cells were positively isolated from splenocytes using Dynabeads FlowComp Mouse CD8^+^ kit (Invitrogen) as described by the manufacturer. The isolated naive CD8^+^ T cells were stimulated with anti-CD3/anti-CD28 activator beads (1:0.8 ratio) for 2 days in AIMV culture medium (Invitrogen) containing 10% FCS, 0.5% penicillin/streptomycin (Invitrogen) and 50 µM β-mercaptoethanol. From day 3 on, an additional mouse recombinant IL-2 (Gibco) was supplemented in the culture media at a final concentration of 100 U/ml allowing cells to expand. T cells were cultured 3–5 days at 37 °C with 5% CO_2_ incubator. Naive CD8^+^ cells were collected immediately after CD8 positive isolation from splenocytes without any stimulation for western Blot and RT-PCR analysis. P815 target cells purchased from ATCC (TIB-64; DSMZ, #ACC1) were cultured in RPMI medium (Invitrogen) containing 10% FCS, 50 U/ml penicillin, 50 µg/ml streptomycin (Invitrogen), and 10 mM HEPES (Invitrogen) at 37 °C with 5% CO_2_.

### Plasmids and antibodies

A pcDNA3.1(+) expression vector with inserted cDNA of the Fwe isoforms from mouse, human and *Drosophila* and respective Fwe mutants were synthesized and purchased from BioCAT. Subcloning into pMAX-mFwe2-mTFP (pMAX vector generated in house [2] was performed using standard cloning procedures (Table 1). Mouse Fwe 1, 2, 3 and 4 (NCBI Reference Sequences NM_001243240.1, NM_029862.4 for mFwe2 and 4, and NM_001243239.1, respectively) and human Fwe cDNA hFWE1-4 (NM_001242370.2, NM_001242369.2, NM_001135775.4 and NM_017586.5, respectively) were subcloned into pMax with the restriction sites EcoRI and XbaI. *Drosophila* Fwe construct dFwe-Ubi-mTFP was made by subcloning mTFP into pMAX-dFwe-Ubi-mRFP plasmid [2] NM_140547.5) using the forward primer 5ʹ-ATGGTGAGCAAGGGCGAGGAG-3ʹ and reverse primer 5ʹ-TTTACTTGTACAGCTCGTC-3ʹ with the restriction sites HindIII and NotI. *Drosophila* LoseA (NM_168633.4) and LoseB (NM_168632.4) were codon optimized for *Mus musculus* [35] and synthesized by Integrated DNA Technologies (Leuven) and subcloned into pMax into the restriction sites ExoRI and XbaI. The mTFP-mFwe2 construct was generated by subcloning mFwe2 into the pMAX-mTFP-VAMP7 plasmid with the restriction sites NheI and SacI [36]. Mouse Fwe2 (YRWL to AAAA), mFwe2 (YARI to AAAA) and mFwe2 (YRWL/YARI to AAAA) were synthesized and subcloned and inserted into the EcoRI/XbaI restriction sites, and mFwe1 (YRWL to AAA) and mFwe1 (Δex3) were subcloned and inserted into the EcoRI/NheI sites of the pMax-mTFP vector.

**Table 1:** Plasmids used in this study

Plasmid/Supplier	Construct	Vector backbone	Comment
#1630	Syb2	pMax	C-terminal mRFP tag
#1762	mFwe2	pMax	C-terminal or N-terminal mTFP tag
#A676	mFwe1	pMax	C-terminal mTFP tag
#A677	mFwe4	pMax	C-terminal mTFP tag
#A678	mFwe3	pMax	C-terminal mTFP tag
#A736	mFwe2(YARI to AAAA)	pMax	C-terminal mTFP tag
#A735	mFwe2(YRWL to AAAA)	pMax	C-terminal mTFP tag
#A737	mFwe2(YRWL/YARI to AAAA)	pMax	C-terminal mTFP tag
#A704	mFwe2(E74A)	pMax	C-terminal mTFP tag
#A715 #A740	mFwe2(Y104A) mFwe2(Y104F)	pMax pMax	C-terminal mTFP tag C-terminal mTFP tag
#A739	mFwe1(Δex3)	pMax	C-terminal mTFP tag
#A738	mFwe1(YRWL to AAAA)	pMax	C-terminal mTFP tag
#A667	hFwe1	pMax	C-terminal mTFP tag
#A669	hFwe2	pMax	C-terminal mTFP tag
#A671	hFwe3	pMax	C-terminal mTFP tag
#A673	hFwe4	pMax	C-terminal mTFP tag
#A700	dUbi	pMax	C-terminal mTFP tag
#A674	dLoseA	pMax	C-terminal mTFP tag
#A675	dLoseB	pMax	C-terminal mTFP tag
#A715 #A720	dUbi(Y109A) mFwe2	pMax pMax	C-terminal mTFP tag C-terminal mTFP tag/ N-terminal mScarletI tag
Addgene/ #137,805	ER-mScarletl	pEGFP-N1	

Point mutations were introduced with the Q5 Site-Directed Mutagenesis Kit (BioLabs) with the corresponding primer for mFwe2(E74A) (5ʹ GTTGTTGTGCGCTGCTCCCTTCTG 3ʹ and 5ʹ AG GATGAAGGCGTTCATG 3ʹ), mFwe2(Y104A) (5ʹ GGCTGTCTTCGCATGCGGGATGGCCAT-CGTCC 3ʹ and 5ʹ TTCTGCCAGGAGCGCAGC 3ʹ), mFwe2(Y104F) (5ʹ GGCTGTCTTCTTCT-GCGGGATGG 3ʹ and 5ʹ TTCTGCCAGGAGCGCAGC 3ʹ) and dUbi(Y109A) (5ʹ AGCAGGTC TTGCAATTGCAATGGCC 3ʹ and 5ʹCGGAAATACAG AGGCTTAC 3ʹ).

All constructs were tagged with the mTFP fluorescent tag at the N- or C-terminus with the linker sequence 5ʹ-AGCGGTGGGAGCGGCGGAAGCGGCGGTTCT-3ʹ, encoding for amino acids S-G-G-S-G-G-S-G-G-S between the gene and fluorescent tag. All constructs were confirmed by sequencing with respective forward and reverse primers by Microsynth Seqlab.

The antibodies used in this work are described in detail in Table 2. All the antibodies except the anti-Fwe antibody (an in-house-generated antibody) are commercially available. Syb2-mRFP [11] was subcloned into pMAX vector backbone with the BamHI/NotI restriction sites.

**Table 2:** Details of antibodies used in this study

Antibody	Supplier/identifier/Reference	Host	Working dilution
Primary antibodies			
Anti-mouse Fwe (affinity purified)	Generated in house/(Chang et al. 2018)	Rabbit, pc	1:100 (ICC) 1:3000 (WB)
Anti-mouse Fwe (precleaned against Fwe KO spleen)	Generated in house/in this study	Rabbit, pc	1:10 (Immuno EM) 1:500 (WB)
Anti-Granzyme B-Alexa647	BioLegend/Clone GB11	Rabbit, pc	1:200(ICC)
Anti-GAPDH	Cell signaling/Clone 14C10	Rabbit, mc	1:5000 (WB)
Anti-Tubulin	Abcam ab4047	Rabbit, pc	1:5000 (WB)
Anti-tRFP	Evrogen/#AB233	Rabbit, pc	Rabbit, pc 1:10 (Immuno EM)
Anti-RFP	Genway/#3BF397	Rabbit, pc	1:1000 (ICC) 1:5000 (WB)
Anti-RFP-Alexa647	Genway/#3BF397 labeled with Alexa647 (Chang et al., 2016)	Rabbit, pc	1:100 (live cell endo-cytosis)
Anti-DsRed	Aviva System Biology/OARA01891	Rabbit, pc	1:1000 (WB)
DAPI	Thermo Scientific/#10116287		1:10,000 (ICC)
Anti-CD8a-Alexa647	Biolegend/#100724	Rat, mc	1:200 (ICC)
Anti-CD62L-FITC	BD Biosciences/#553150	Rat, mc	1:400 (FACS)
Anti-CD44-APC	eBioscience/#17-0441-82	Rat, mc	1:400 (FACS)
Anti-CD25-PE	BD Biosiences/#553866	Rat, mc	1:200 (FACS)
Anti-mouse Tfrc (CD71, purified)	Biolegend/Clone RI7217	Rat, mc	1:200 (FACS)
Anti-IgG2a (Isotype control)	R&D systems/Clone 54447	Rat, mc	1:200 (FACS)
Anti-mouse CD3ε	BD Pharmingen/Clone 145-2C11	Hamster, mc	3:100 or 1:100 (glass coating)
Anti-mouse LAMP1- Alexa647	Biolegend/Clone 1D4B	Rat, mc	1:200 (live cell endo-cytosis)
Secondary antibodies			
Anti-rat IgG2a, FITC	Novus Biologicals/#NB7124	Goat, pc	1:400 (FACS)
Anti-rabbit IgG, F(ab’)2, HRP	Millipore/#AQ132P	Goat, pc	1:10000 (WB)
Anti-rabbit IgG (H + L), HRP	Thermo Fisher Scientific/#31460	Goat, pc	1:10000 (WB)
Anti-rabbit IgG (H + L) 10 nm gold labeled	Aurion/#810.011	Goat, pc	1:30 (Immuno EM)

*mc* monoclonal, *pc* polyclonal

### Flow cytometry and cell sorting

For flow cytometry analysis, 1 × 10^6^ naive and activated day 3‒5 WT and Fwe KO CD8^+^ T lymphocytes were resuspended in D-PBS (Gibco) and incubated in the dark for 30 min on ice with cell surface-specific FITC-, APC- or PE-conjugated antibodies against CD44, CD62L and CD25 (Table 2). The viable T lymphocytes were gated on the basis of their cell size and granularity. Data were acquired by using a BD FACSAria III analyzer (BD Biosciences) with BD FACSDiva^TM^ software 6.0. The data were analyzed with FlowJo v10.0.7 software.

For the transferrin receptor 1 (TfR1) flow cytometric internalization assay, day 5 WT and Fwe KO CTLs were washed with ice-cold PBS and incubated with purified rat anti-mouse TfR1 for crosslinking or Rat IgG2a isotype control for 30 min on ice (Table 2). Cells were then washed and resuspended in cold PBS, and an aliquot was stained with a goat anti-rat IgG2a-FITC antibody to determine baseline TfR1 expression. The remaining cells were incubated at 37 °C for 15 or 30 min, and internalization was stopped by incubating the cells on ice and washing them with ice-cold PBS. The cells were stained with a fluorescent-coupled secondary antibody, followed by flow cytometry analysis. For the control, WT CTLs were pretreated with 15 μM pitstop2 (ab120687; Abcam; 30 mM stock) or pitstop2 negative control (ab120688; Abcam; 30 mM stock) for 3 min at room temperature in serum-free AIMV, followed by normal staining procedure with TfR1 staining. Percentage of internalized TfR1 was calculated as follows: (MFI at zero min - MFI at specified time point) × 100/MFI at zero min, where MFI stands for mean fluorescent intensity.

For cell sorting, 8 × 10^6^ Fwe KO mCTLs on day 3 were transfected with 10 µg pMax-mFwe2-mTFP by electroporation (Mouse T Cell nucleofector Kit, Lonza). Cells were incubated for 12 h at 37 °C with 5% CO_2_, collected, centrifuged at 900 rpm for 10 min and resuspended in 800 µl D-PBS for cell sorting. Gating was done against non-fluorescent WT mCTL. 0.2–0.5 × 10^6^ sorted cells were collected, resuspended in IMDM culture medium supplemented with 10% FCS, 50 U/ml penicillin, 50 µg/ml streptomycin and 50 µM β-mercaptoethanol and cultured for 2 h to recover. Cells were then collected and resuspended in AIMV medium at a density of 1 × 10^6^ cells/ml for electron microscopy.

### RT-PCR

Total RNA from naive and activated WT and Fwe KO CTLs was extracted with TRIzol (Thermo Fisher Scientific) and reverse transcribed with SuperScript^TM^ II (Thermo Fisher Scientific) using hexamer random primers. Semi-quantitative PCR was performed using intron-spanning primers: mFwe1 (forward 5ʹ-ATCTCTGGACTCTTCAACT-3ʹ and reverse 5ʹ-GCAAATGCTGATGTACACA-3ʹ), mFwe2.1 (forward 5ʹ-ATCTCTGGACTCTTCAACT-3ʹ and reverse 5ʹ-GAGTTCATCCCATCCATAG-3ʹ), mFwe2.2 (forward 5ʹ-ATCTCTGGACTCTTCAACT-3ʹ and reverse 5ʹ-ACTCATCTAATAAGCCACTG-3ʹ), mFwe3 (forward 5ʹ-ATCTCTGGACTCTTCAACT-3ʹ and reverse 5ʹ-TTAGAGGGAAATGGTGTTTCT-3ʹ), mFwe4 (forward 5ʹ-TTGCTAAATCCTGGGTGTCC-3ʹ and reverse 5ʹ-TCACAGTTCCCCCTCGAAAG-3ʹ), mFwe5 (forward 5ʹ-TATTTTGAGGGGTTTTGCAG-3ʹ and reverse 5ʹ-CACAACAACAGGATGAAGG-3ʹ) and SDHA (forward 5ʹ-AGATGGGAAGATTTATCAGC-3ʹ and reverse 5ʹ-GAGACACAACATCTCTTGA-3ʹ). The expression levels of the transcripts were normalized to *SDHA*. For positive control, the procedure described above was also performed for WT adult mouse whole brains. Genomic DNA was used as negative control.

### Western blot and Native PAGE

For western blotting, cell lysates were separated by SDS-PAGE on precast 10% Bis-Tris gels with MES running buffer (NuPage, Invitrogen). Proteins were then transferred to 0.2 µm pore-size nitrocellulose membrane and blocked with 5% non-fat dry milk powder in TBS buffer containing 20 mM Tris, 0.15 M NaCl, and 0.05% Tween 20, pH 7.4 for 1 h at RT (20±2 °C). For quantitative western blots protein concentrations were measured using Pierce^TM^660 reagent (Thermo Fisher Scientific) or Bradford (Bio-Rad). The immunoblots were analyzed with anti-Fwe (1:1000 or 1:500) (affinity-purified or affinity-purified and precleaned against spleen of Fwe KO mouse) or anti-DsRed to detect mTFP (1:1000), anti-GAPDH (1:1000) and horseradish peroxidase (HRP)-conjugated goat anti-rabbit secondary antibodies (1:10000) (Table 2). For reprobing, western blot membranes were stripped in stripping solution (Invitrogen) for 15 min at RT. Finally, the blots were developed using enhanced chemoluminescence reagents (SuperSignal West Dura Chemoluminescent Substrate; Thermo Fisher Scientific) and imaged by gel documentation (FluorChem M system, ProteinSimple).

For native PAGE, 4–8 × 10^6^ mCTLs were lysed in modified RIPA buffer (50 mM Tris HCl, pH 7.5, 50 mM NaCl, 1% Triton-X-100 and 1 mM EDTA pH 8.0, containing proteinase inhibitors (Complete Mini EDTA-free (Roche) and 2 mM Pefabloc SC). After 30 min solubilization at 4 °C, lysates were centrifuged (16,000 × g, 30 min, 4 °C). The supernatant was measured by Pierce^TM^ 660. Lysates were mixed with NativePAGE sample buffer (4x, Invitrogen) and 5% G-250 Additive (Invitrogen) and loaded on 4–16% precast Novex 4–16% Bis-Tris gels (Invitrogen) with the NativePAGE running buffer supplemented with 0.02% and 0.002% G-250 Additive (Invitrogen) [37]. A NativeMark^TM^ unstained protein ladder (Invitrogen) was used as the molecular mass standard. Proteins were transferred to a 0.2 µm pore size PVDF membrane via standard western blotting. The immunoblots were analyzed with anti-DsRed and anti-rabbit HRP-conjugated goat secondary antibodies as described above.

### Total internal reflection fluorescence microscopy (TIRFM)

The TIRFM setup (Visitron Systems GmbH) was based on an IX83 (Olympus) equipped with a UAPON100XOTIRF NA 1.49 objective (Olympus), solid-state excitation lasers at 488 nm, 561 nm and 647 nm, an iLAS2 illumination control system (Roper Scientific SAS), a QuantEM 512SC camera (Photometrics) and a filter cube containing Semrock (Rochester) FF444/520/590/Di01 dichroic and FF01-465/537/623 emission filters. The setup was controlled by Visiview software (version 4.0.0.11, Visitron GmbH). For TIRFM, day 4 bead-activated CTLs isolated from Fwe KO mice were transfected with mFwe2-mTFP or the mScarletI-mFwe2-mTFP isoform. 12–16 h after transfection, cells were resuspended in 30 µl of extracellular buffer (2 mM HEPES, 140 mM NaCl, 4.5 mM KCl, and 2 mM MgCl_2_) and allowed to settle for 2–3 min on anti-CD3ε antibody (30 μg/ml, Table 2) coated coverslips. Cells were then perfused with extracellular buffer containing high concentrations of calcium (10 mM glucose, 5 mM HEPES, 140 mM NaCl, 4.5 mM KCl, 2 mM MgCl_2_ and 10 mM CaCl_2_) to stimulate CG secretion. Cells were imaged for 10 min at room temperature with 488 nm excitation wavelength. Acquisition frequency was 10 Hz and the exposure time was 100 ms. Acquired images and time-lapse series were analyzed using ImageJ v1.46 software.

## Confocal imaging

For endocytosis rescue experiments, activated day 5 WT and Fwe KO CTLs were transfected with pMAX-Syb2-mRFP plasmid as a CG marker (Table 1). After 12–16 h, 0.1 × 10^6^ cells in 150 μl AIMV medium supplemented with 10 mM HEPES were allowed to settle down onto glass-bottom dishes (IBIDI) coated with 0.01% poly-L-ornithine (Sigma). 0.02 × 10^6^ P815 target cells in 50 μl AIMV containing 2 μl anti-CD3ε antibody (stock-1 mg/mL) and 2 μl of Alexa647 conjugated anti-RFP antibody (stock ~1 mg/ml) were added to visualize Syb2 endocytosis (Table 2). For Fwe rescue experiments, CTLs from Fwe KO mice were transfected with respective isoforms or mutants cloned in pMAX-mTFP plasmid vector (Table 1).

Live imaging was performed at 37°C using a Zeiss laser scanning microscope 780 (LSM 780). Movies and images were acquired with a 63x Plan-Apochromat immersion objective with an NA of 1.4. Wavelengths for exciting samples (488, 561 and 633 nm) were used with a pinhole size of 1 arbitrary unit (AU). Confocal z-stacks were taken with a step size of 1 μm and scanning speed of 1 s/plane. Maximum intensity projections were calculated from the collected data and analyzed as a function of time (t). Further analyses were done using ImageJ software. The analysis for the accumulation of endocytosed CG at the IS over time was performed by calculating the percentage of total fluorescence of the anti-RFP-Alexa647 antibody at the IS using the formula (fluorescence at the IS/fluorescence in the entire cell × 100) of maximal image projection images. The CTL was identified and a rectangular ROI was placed with one end tangential to the target cell. The ROI was then divided into three equal parts. The IS was defined as the third of the ROI adjacent to the target cell. Typically, the ROI area was approximately 20–25% of the total ROI area.

Analyses of fluorescence intensity for the different Fwe-mTFP fusion constructs were done by marking a ROI over the entire cell and over the nuclear area. The intensity of the selected ROIs was measured using ImageJ software and the cytoplasmic fluorescence of the cell was calculated using the formula:

$${\text{Fluorescence intensity }} = \frac{{\left( {{\text{Intensity of total cell }}{-}{\text{ Intensity in nucleus}}} \right)}}{{\left( {{\text{Area of total cell }}{-}{\text{ Area of nucleus}}} \right)}}$$

### Anti-RFP antibody coupling to Alexa fluorophore

To visualize Syb2 endocytosis, Alexa 647 coupled anti-RFP and antibody was used to track the endocytic events. The coupling procedure was followed by the instruction of the manufacture (Invitrogen, Alexa Fluor Antibody Labeling Kit). In brief, 100 μl of a complete stock of anti-RFP antibody (1 mg/ml) was used for one conjugation reaction. 10 μl of sodium bicarbonate buffer (1 M stock in dH_2_O) was added to the anti-RFP antibody to alkalinize pH for more efficient coupling for succinimidyl esters or TFP esters on Alexa fluorophores. Then 110 μl antibody was transferred to the vial of reactive Alexa dye provided in the kit. The coupled antibody mixture was then incubated for 1 h at RT (20 ± 2 °C) on a rotator and transferred into the pre-prepared resin column. The eluate was centrifuged for 3 min at 1100 × g and used for the experiments.

### Immunocytochemistry and structured illumination microscopy (SIM)

**Fig. 1:**
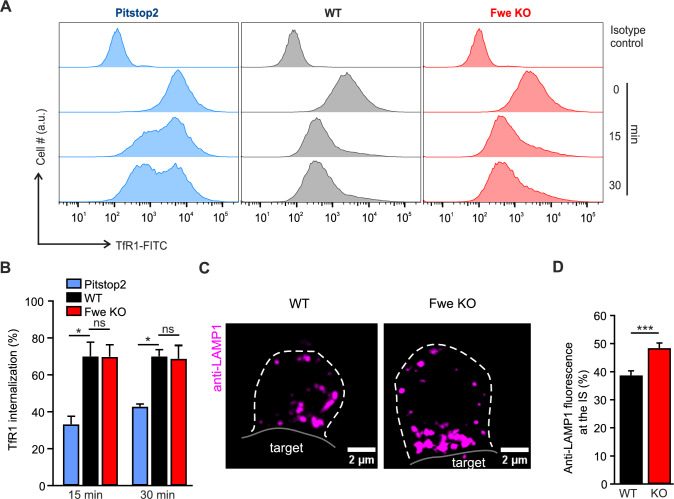
Loss of Flower does not reduce TfR1 endocytosis but affects LAMP1 endocytosis. **A** Representative flow cytometric histogram showing TfR1 staining of CTLs activated for five days. TfR1 staining was measured at 0 min, 15 min and 30 min in WT treated with pitstop2, WT and Fwe KO CTLs. The isotype control (upper row) was used for baseline gating. **B** Quantification of the pooled mean fluorescence intensity of TfR1 internalization in pitstop2-treated, WT and Fwe KO cells. Error bars represent mean ± SEM (N = 3); One-way Analysis of Variance followed by multiple comparison (Dunnett’s), *p < 0.05, ns p > 0.05. **C** Representative SIM images of LAMP1 internalized in WT and Fwe KO CTLs that were in contact with P815 target cells. CTL and target cells were fixed after 30 min of incubation with anti-LAMP1-647 (magenta) antibody. Scale bar, 2 µm. **D** Quantification of the fluorescence signal of endocytosed LAMP1 at the immunological synapse (one-third of the cell volume) *versus* the whole cell in Fwe KO cells in comparison to WT cells. Data given as mean ± SEM (N = 2, n = 34 for both conditions). Data significance was analyzed by Student's t-test, two-tailed ***p < 0.001

To study the localization of Fwe at the plasma membrane, day 4 activated Fwe KO CTLs were transfected with mouse isoforms in pMAX-Fwe-mTFP background and with ER-mScarletl plasmid (Addgene) (Table 1). After 12–16 h at 37 °C, the cells were washed in AIMV medium, and bead stimulated for 1 h at 37 °C. Cells were kept on ice and fixed in ice-cold 4% PFA for 20 min after beads were removed. Cells were treated with permeabilizing solution (0.1% Triton-X-100 in PBS) for another 20 min, followed by 30 min blocking solution (2% BSA prepared in permeabilizing solution) and stained with anti-CD8a-Alexa647 (Biolegend) for 30 min on ice. Finally, cells were mounted with Mowiol based mounting medium and observed at SIM microscope (Zeiss Elyra PS.1). For LAMP-1 endocytosis experiment shown in Fig. 1C and D, day 5 CTLs were co-cultured with P815 target cells in the presence of 10 μg/ml anti-CD3ε antibody and 2.5 μg/ml anti-LAMP1-Alexa647 antibody in RPMI culture media on a poly-L-ornithine coated coverslip. Cells were incubated for 30 min at 37 °C with 5% CO_2_ allowing LAMP-1 antibody to endocytose. To compare Flower-mediated endocytosis in stimulated and unstimulated T cells, Flower KO cells were placed on poly-L-ornithine- or anti-CD3ε-coated coverslips for 40 min in the presence of 2.5 μg/ml anti-LAMP-1-Alexa647 (Biolegend) or 5 μg/ml anti-transferrin receptor-Alexa488 (Novus Biologicals). Cells were then fixed with 4% PFA and stained with 1 μg/ml WGA-Alexa594 in PBS to mark the plasma membrane. To observe the localization of expressed wild-type Fwe2 and its mutants, day 5 effector Flower KO cells were co-transfected with Syb2-mRFP and Fwe constructs. These KO cells were co-incubated with P815 in the presence of anti-RFP-Alexa647 and 1 μg/ml anti-CD3ε antibodies to label Syb2 endocytic events for 30 min at 37 °C. Cells were then fixed with 4% PFA, washed twice with D-PBS and mounted for SIM imaging. The total volume of the cells was taken in the images (about 5–10 μm thickness) for analysis. Z-stacks were acquired with a step size of 200 nm. All images were acquired with a 63 × Plan-Apochromat (NA 1.4) and then processed to obtain higher resolutions by Zen 2012 (Zeiss). Analysis was performed as described for the anti-RFP-Alexa647 uptake experiments.

### Electron microscopy

To localize endogenous Fwe protein, mouse CTLs were isolated from WT and Fwe KO mice as described above. Additionally, Fwe KO CTLs were electroporated with pMAX-mFwe2-mTFP. After cell sorting, as described above, cells were added to flat specimen carriers containing 0.01% poly-L-ornithine and 30 µg/ml anti-CD3ɛ antibody coated 1.4 mm sapphire discs. The cells were allowed to settle down for 5–10 min to form an artificial immunological synapse and were vitrified in a high-pressure freezing system (Leica EM PACT2/RTS). Vitrified samples were transferred into the pre-cooled (−130 °C) freeze-substitution chamber of an AFS2 (Leica). The temperature was increased from −130 to −90 °C for 2 h. Cryo-substitution was performed at −90 °C to −70 °C for 20 h in anhydrous acetone and at −70 to −60 °C for 24 h with 0.3% (w/v) uranyl acetate in anhydrous acetone. At −60 °C, the samples were infiltrated with increasing concentrations (30, 60 and 100%; 1 h each) of Lowicryl (3:1 K11M/HM20 mixture). After 5 h of infiltration with 100% Lowicryl, samples were UV polymerized at −60 °C for 24 h and for additional 15 h while temperature was raised linearly to 5 °C. Samples were kept in the dark at 4 °C until further processing. After removing the carriers and the sapphire discs, 100 nm ultrathin sections were cut using an EM UC7 (Leica) and collected on pioloform-coated nickel grids (Plano).

Postembedding immunogold electron microscopy was done according to the user’s manual (Aurion). In brief, the ultrathin sections were first treated with blocking solution (Aurion) for 30 min at RT to block nonspecific protein binding sites. Then, the primary rabbit polyclonal anti-Fwe antibody (25 µg/ml) or anti-tRFP antibody (1:10) was diluted in incubation solution (PBS, pH 7.4 with 0.1% BSAc (Aurion)) and applied for 2 h. After several wash steps with incubation solution, the primary antibody was detected with goat anti-rabbit secondary antibody conjugated to 10 nm gold particles (Aurion). Following multiple wash steps with PBS, immune complexes were fixed with 2% glutaraldehyde in PBS for 15 min at RT. After contrasting with uranyl acetate and lead citrate sections were analyzed with a Tecnai12 Biotwin electron microscope (Thermo Fisher).

### Imaging data analysis and statistical analysis

All images were analyzed with ImageJ or FIJI version 15 and above (National Institute of Health). All statistical tests were performed with Igor (Igor Pro 6.37) or SigmaPlot 14.5, and data are represented as mean ± SEM (or SD when specified). The statistical tests are indicated in each figure legend. The Shapiro-Wilk-test was used to confirm normality prior to application of the Student’s t test. Non-parametric Mann-Whitney U-tests were performed for small samples. For multiple comparisons, we used one-way ANOVA followed by Dunnett’s post hoc tests when data were normally distributed. If data distribution was non-normal, we used the Kruskal-Wallis Test with Dunn’s multiple comparisons instead. Graphs show mean values ± SEM unless otherwise described.

## Results

### Loss of Flower does not affect transferrin receptor 1 endocytosis but reduces the recycling of LAMP1 at the immune synapse

We assessed Fwe function in clathrin-mediated endocytosis by comparing the uptake of TfR1 in CTLs treated with pitstop2, an inhibitor of clathrin-mediated endocytosis [16], to that in Fwe KO CTL and wild type (WT) CTL. TfR1 uptake was assessed using FACS analysis with an FITC-labeled TfR1-antibody (Fig. 1A). In the pitstop2 treated CTLs, an accumulation of FITC signal at the cell surface even after 30 min incubation indicates a reduction in endocytosis. In WT and Fwe KO cells, the FITC fluorescence was reduced over time, indicating ongoing endocytosis. TfR1-FITC internalization was similar in control and Fwe KO CTL (near 70% at 15 and 30 min) while it was significantly lower in pitstop2 treated WT cells (Fig. 1B). We also investigated the role of Fwe in TfR1 endocytosis with SIM imaging in anti-CD3ε stimulated and unstimulated cells. Under both conditions, we observed abundant TfR1 uptake in WT and Fwe KO cells extending the result of the FACS analysis of stimulated cells (Fig. S1A, B). These data show that Fwe is not involved in TfR1 endocytosis response in classical clathrin-mediated endocytosis.

Since it was shown that Fwe promotes Syb2 endocytosis, we investigated whether it has a general role in CG membrane protein uptake by studying LAMP1 endocytosis [2]. LAMP1 is a lysosomal membrane protein regularly used as a CG marker [17]. In resting CTL, surface expression of LAMP1 is similar in WT and Fwe KO CTL [2]. However, LAMP1 is not only associated with CG but also to large extend with other lysosomes [18]. The CG fraction of LAMP1 becomes exposed to the extracellular surface of CTL following CG fusion only after T-cell receptor engagement (Fig. S1C) [9]. To get exclusively access to this exocytosed LAMP1, we applied fluorescent anti-LAMP1-Alexa647 antibody to CTLs in contact with P815 target cells. Upon binding with LAMP1, this antibody then appears in the cytosol of the CTL, allowing imaging of LAMP1 endocytosis. Cells were fixed after 30 min of co-culture and the fluorescence was visualized. The accumulated LAMP1 fluorescent signal at the IS in KO CTLs demonstrates an endocytic deficiency in KO CTLs (48.4% ± 1.83) compared to WT cells (38.7% ± 1.64, p = 0.0002) (Figs. 1C, D and S1D). Taken together, these data indicate that Fwe, though not required for general clathrin-mediated endocytosis, promotes endocytosis of CG components.

### Mouse Flower 2 is localized on vesicular structures and is delivered to the plasma membrane upon T cell target cell stimulation

**Fig. 2:**
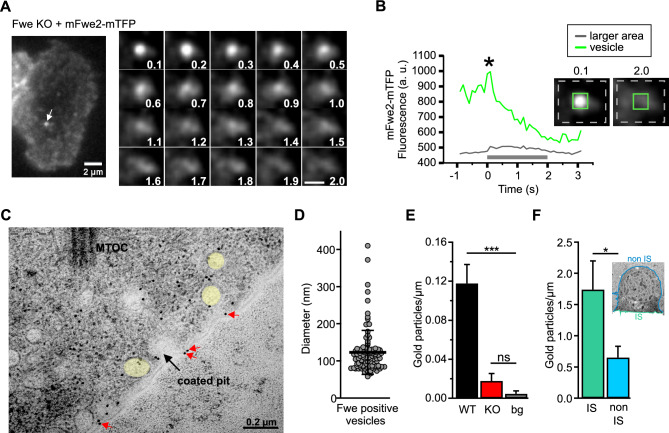
Mouse Flower 2 is localized on small vesicles that fuse with the plasma membrane upon T cell stimulation. **A** Live cell TIRFM imaging of mFwe2-mTFP expressing Flower KO CTL. Snapshot images of an exemplary cell and a Flower fusion event. Overall, 20 fusion events were measured in 11 cells expressing either mFwe2-mTFP or mScarletI-mFwe2-mTFP. The images were recorded at 10 Hz. Scale bar 2 µm and 1 µm, respectively. **B** Fluorescence intensity time course of the mFwe2-mTFP-positive vesicle shown in **A**. The green line shows the fluorescence intensity at the vesicle (insert with green square), whereas the gray line corresponds to the intensity of a larger membrane area (insert with stippled gray rectangle). Fusion occurs when the green line shows maximum fluorescence intensity (*). Its decay indicates dispersion of the mFwe2-mTFP in the plasma membrane following fusion of the vesicle. Accordingly, a slight increase in fluorescence is observed in the surrounding plasma membrane. The gray bar corresponds to the time of the snapshots shown in **A**. **C** A representative electron micrograph showing part of the immunological synapse of a CTL expressing mFwe2-mTFP. Post-embedding immunogold electron microscopy was done on activated mCTLs settled on anti-CD3ε coated sapphire discs. The protein was labeled with the primary antibody, anti-tRFP against mTFP, and with a secondary gold-conjugated antibody (10 nm). The microtubule organizing center (MTOC) is present, as are Fwe-positive vesicles (yellow). Flower was identified on the plasma membrane (red arrows) and on a coated pit (black arrow). Scale bar, 0.2 µm. **D** Quantitative analysis of the diameter of immunogold-labeled Flower positive vesicles as shown in **C**. Data given as mean ± SEM (N = 4, n_cells_ = 14, n_vesicles_ = 99). **E** Quantitative analysis of endogenously expressed Flower protein in WT and KO mCTLs localized on the plasma membrane (shown in **C**, red arrows). Post-embedding, immune electron microscopy was done with primary anti-Flower antibody and 10 nm secondary goat anti-rabbit gold antibody on ultrathin sections cut parallel to the sapphire disc. For background control (bg) sections were stained without primary antibody. Gold particles were counted per µm. Data given as mean ± SEM. Kruskal–Wallis One-way Analysis of Variance on Ranks followed by multiple comparison (Dunn’s) was done. (N = 3; WT n = 19, KO n = 18, bg n = 21; ***p < 0.001, not significant (ns)). **F** Quantitative analysis of overexpressed mFwe2-mTFP protein localized on the plasma membrane. Post-embedding immunogold electron microscopy was done as described in **C** with primary anti-Flower antibody and 10 nm secondary goat anti-rabbit gold antibody on ultrathin sections cut vertical to the sapphire disc. Inset shows a representative image of an activated CTL, settled on anti-CD3ε coated sapphire disc with marked immunological synapse (IS, green) and non IS regions (blue). Gold particles localized on the plasma membrane, as shown in **C**, were counted per µm (mean ± SEM (N = 3; non IS n = 18)). Data significance was analyzed by Mann–Whitney Rank Sum Test (*p < 0.05)

Due to the discrepancy between the impact of Fwe on TfR1 and LAMP1 recycling in CTLs with and without target contact, we investigated whether Fwe is differentially localized at the PM in a stimulation-dependent manner. Fwe has been reported to be present on synaptic vesicles in *Drosophila* and to traffic to the plasma membrane [1]. In mouse CTL, Fwe also appears on small vesicular structures. To visualize these Fwe-positive vesicles near the synapse, Fwe KO CTLs were transfected with full-length Fwe constructs (Fwe2-mTFP and mScarletI-Fwe2-mTFP) and then applied to anti-CD3ε pre-coated coverslips to facilitate synapse formation. Fwe positive vesicles were then observed with real-time TIRFM imaging. In average, we observed the fusion of about 2 to 3 Fwe-positive vesicles per secreting cells. The granule shown in Fig. 2A reached the plasma membrane at 0.1 s and subsequently fused with a gradual dispersion of the membrane-bound Fwe-mTFP over the following two seconds (Fig. 2A, right panel). Figure 2B shows a time sequence of the mean fluorescence measured from a region of interest (ROI) placed at the position of the vesicle as it reached the plasma membrane, and of the mean fluorescence of the entire frame. Though the fluorescence at the point of fusion rapidly decayed, the total brightness changed little in the two seconds following fusion (*), indicating that the Fwe-mTFP fluorescence remained at the plasma membrane while diffusing away from the point where fusion occurred.

We further investigated the localization of the Fwe protein in CTLs via immunogold labeling with an anti-Fwe antibody by electron microscopy. The gold particles labeled vesicular compartments (highlighted in yellow) and the plasma membrane in Fwe KO CTL expressing mFwe2-mTFP (Fig. 2C) and the endogenous localization of the Fwe protein (Fig. S2). The average diameter of the immunogold-marked Fwe-positive vesicles was 122.92 ± 5.95 nm (Fig. 2D). We compared the amount of endogenous Fwe by counting the bound gold particles at the plasma membrane of Fwe KO, WT CTL and a background control (without primary antibody). WT CTL displayed a significant higher abundance of 10 nm gold particles at the PM than KO CTL (Fig. 2E). In mFwe2-mTFP overexpressing cells, we compared the density of gold particles at the IS to that of the rest of the cell. CTLs stimulated with anti-CD3ε-coated coverslips showed significant enrichment of Fwe protein at the plasma membrane of the synapse (Fig. 2F). These results show that Fwe reaches the plasma membrane upon stimulation via fusion of Fwe-positive vesicles and that this occurs predominantly at the IS.

### Four Flower transcripts are expressed in mouse cytotoxic T lymphocytes at different days of activation

**Fig. 3:**
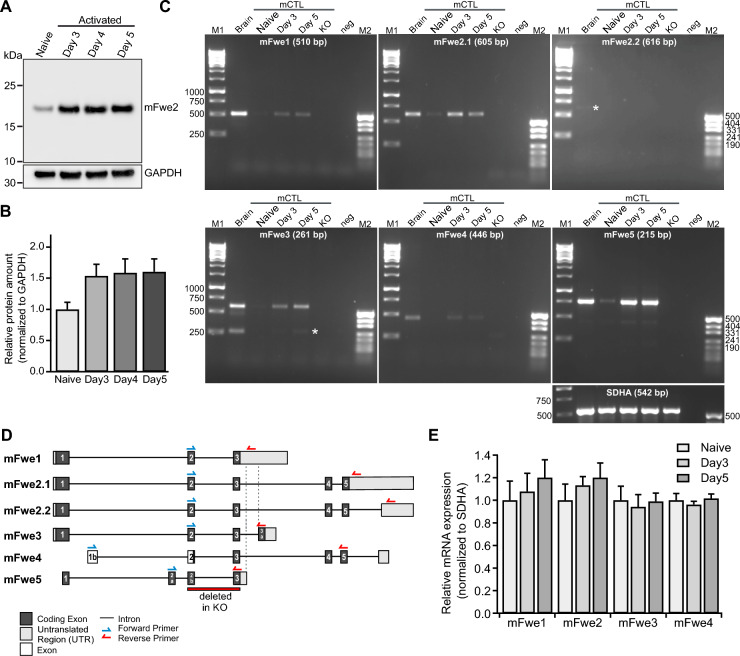
Flower expression in mouse CTL at different days of activation. **A** Representative western blot of Flower protein expression levels in homogenates of naive and stimulated mouse WT CTL of day 3, day 4 and day 5 (20 µg per lane). The loading control was GAPDH. **B** Quantitative analysis of the western blots shown in **A**. Flower expression was normalized to the expression level of *GAPDH*. The data represent means ± SEM from four mice. **C** RT-PCR analysis of mFwe1, mFwe2.1 and 2.2, mFwe3, mFwe4 and mFwe5 transcripts (Transcript: ENSMUST00000114004.8, ENSMUST00000114007.8, ENSMUST00000114006.8, ENSMUST00000114005.9, ENSMUST00000114003.2, ENSMUST00000133807.2, respectively) in WT mouse CTL at different activation states. Total RNA from adult mouse brains was used as the positive control, and total RNA isolated from mFwe knockout spleens was used as the negative control. White stars show the positions of the expected bands in mFwe2.2 and mFwe3. *SDHA* was used as the loading control. **D** Scheme of primer localization for RT-PCR analyses on the six postulated mouse Flower transcripts mFwe1 (ENSMUST00000114004.8), mFwe2.1 (ENSMUST00000114007.8), mFwe2.2 (ENSMUST00000114006.8), mFwe3 (ENSMUST00000114005.9), mFwe4 (ENSMUST00000114003.2) and mFwe5 (ENSMUST00000133807.2). Exon coding regions are shown in black with numbers, and putative exons are shown in white with numbers. Untranslated regions are shown in gray. The red bar shows the targeted deletion of exon 2 and exon 3 in the mFwe KO mouse. **E** Expression of mFwe1, mFwe2, mFwe3 and mFwe4 in naive, day 3 and day 5 CTL normalized to the expression of the *SDHA* as housekeeping gene. The data represent means ± SD of samples from three mice per condition

The mouse *Fwe* gene encodes five different splice variants, which result in the expression of four protein isoforms (Fig. 3D) (www.ensemble.org, [19, 20]). Mouse Fwe 2 (mFwe2, the full-length form), is prominently expressed and present in naive CTL and at higher levels in cells activated with anti-CD3/anti-CD28 activator beads for three, four and five days (western blot analysis, Fig. 3A, B). We then determined which Fwe isoforms are expressed in mouse CTL at different activation states.

To analyze the transcription of the murine Fwe splice variants in mouse CTL, RT-PCR was performed on total RNA isolated from naive and activated day three and day five CTL (Fig. 3C). The primers for the RT-PCR were designed specifically at characteristic exon–intron borders to identify and differentiate the different splice variants. Figure 3D illustrates the structure of the mouse *Fwe* gene, as well as the five isoforms that can be expressed in mouse CTLs. In addition, the positions of the primers used for PCR are shown. Whole-brain RNA from adult mice was used as a positive control, and total RNA from day five Fwe KO CTL was used as the negative control. The amplified DNA bands had the appropriate size and indicated that mFwe1, mFwe2, mFwe3 and mFwe4 were expressed. mFwe2.2 is barely detectable and generates the same protein as Fwe2.1. The relative mRNA expression was normalized to *SDHA* expression (Fig. 3C, E; N = 3). Activated mFwe KO CTLs were transfected with murine Fwe isoforms Fwe1, Fwe2, Fwe3 and Fwe4 at day five for further experiments.

### Flower KO CTL show similar proliferation and activation state compared to WT cells

Since CTL proliferation is an important indicator of cell integrity [21], we compared the division state and subset transition during activation in Fwe KO and WT CTLs. CSFE staining of WT and Fwe KO CTLs was performed to monitor distinct generations of proliferating cells by dye dilution. Flow cytometry analysis of unstained and CSFE-stained naive, undivided CTLs and their proliferation states at days three, four and five, of WT and Fwe KO mice were analyzed. The result showed no difference between the proliferation states in WT and Fwe KO CTLs (Fig. S3A).

Furthermore, the degree of differentiation of WT and Fwe KO CTLs was determined by FACS (Fig. S3B, C). CD44 and CD62L surface markers were used to define naive (CD44-/CD62L-), central memory (CD44 + /CD62L +), and effector memory (CD44 + /CD62L-) CTL cells [22]. CD25 was used as activation marker. Since all subsequent experiments were performed on day five, CTL subset staining was performed on day five. Activated WT and Fwe KO CTLs presented similar profiles, which consisted mainly of the effector memory population and some remaining central memory cells (Fig. S3B, C; N = 3).

### Mouse Flower isoforms differ in their ability to restore Synaptobrevin2 endocytosis.

**Fig. 4:**
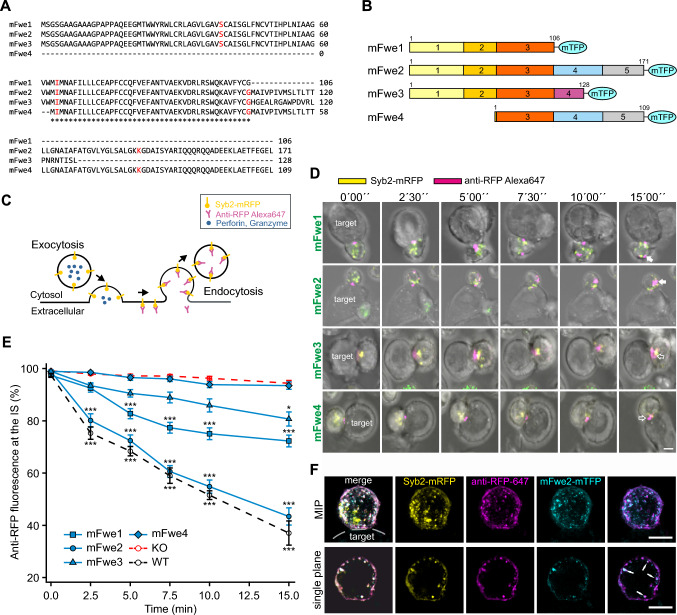
Mouse Flower isoforms differ in Synaptobrevin2 endocytosis efficiency. **A** Sequence alignment of mouse Flower (mFwe) isoforms 1 to 4 using Clustal Omega. The exon/intron borders are shown in red. (*) Indicates conserved amino acids in exon 3. **B** Murine Flower isoform constructs. The exon structures of the four mouse isoforms are shown, as are the number of amino acids and the linker region at the C-terminus with the coupled fluorophore mTFP. **C** Schematic drawing of the experiment shown in **D**. **D** Series of images of Fwe KO CTLs expressing Syb2-mRFP (yellow) and each isoform of mFwe-mTFP that were acquired over 15 min. CTLs were conjugated with P815 in the presence of anti-RFP-Alexa647 antibody (magenta) in the medium. Images were acquired at 0, 2.5, 5, 7.5, 10 and 15 min. White arrows mark endocytosed organelles, and open white arrows indicate organelles at the IS. Scale bar: 5 μm. **E** Quantitative analysis of endocytosed Syb2-mRFP signals (based on anti-RFP-Alexa647 antibody fluorescence) at the immunological synapse (IS) for mFwe1-4, as shown in **D**, in comparison to WT and Flower KO mouse CTL. Time zero was defined as the appearance of the first anti-RFP-Alexa647 signal at the IS. Data given as mean ± SEM; Kruskal–Wallis One-way Analysis of Variance on Ranks followed by multiple comparison (Dunn’s) was done *versus* Fwe KO as control (*p < 0.05, **p < 0.01, *** p < 0.001, KO n = 19 (N = 4); WT n = 16, mFwe1 n = 21, mFwe2 n = 16, mFwe3 n = 18, mFwe4 n = 19 (N = 3)). **F** SIM images of a Fwe KO CTL co-expressing Syb2-mRFP and mFwe2-mTFP in contact with a P815 target cell. Anti-RFP-Alexa647 antibody was applied in the medium to label endocytic Syb2. Cells were fixed after 40 min to allow synapse formation and endocytosis to occur. MIP images and single stack images of the same cell are shown to demonstrate the precise co-localization of mFwe2 and endocytic Syb2 (white arrows)

To ascertain the domain of Fwe required to promote endocytosis of CG membrane proteins, we examined the ability of naturally occurring Fwe isoforms to rescue endocytosis in Fwe KO cells. We started by evaluating the mouse isoforms Fwe1-4, whose sequence alignments are shown in Fig. 4A. Each of these proteins was expressed as a pMAX-Fwe-mTFP fusion protein in murine Fwe KO CTLs (Fig. 4B). We used Syb2, the v-SNARE mediating CG fusion [11], as a highly specific membrane protein marker for CG endocytosis [13]. The experiment was performed as follows: Fwe KO CTLs were transfected with Syb2-mRFP together with the different Fwe constructs and placed in contact with P815 target cells to induce CG exocytosis. The mRFP tag is located in the granule lumen and is exposed to the extracellular space after CG fusion (Fig. 4C). The cells were then treated extracellularly with an Alexa647-labeled anti-RFP antibody, which readily binds specifically to the mRFP exposed at the cell surface. Following endocytosis, the fluorescently tagged anti-RFP-Alexa647 was internalized and distributed within the cytoplasm (Fig. 4C). These endocytosed Syb2 vesicles are visualized as double labeled spots. Cross reactivity of the anti-RFP antibody with mTFP was avoided due to the localization of the fluorescent protein. While the mRFP is exposed to the extracellular space upon CG exocytosis, the mTFP attached to Fwe remains intracellular (Fig. S4A). We followed this process by live-cell confocal imaging over a period of 15 min.

Time-lapse snapshots of Fwe KO CTLs expressing the four murine isoforms tagged with mTFP in contact with a P815 target cell (Fig. 4D) show the accumulation of anti-RFP-Alexa647 at the contact zone, the IS. The first occurrence of endocytic Syb2 is indicated as time 0′00’’. Over time, endocytosis of Syb2 was observed as fluorescent puncta moving from the IS into the cell (Fig. 4D). We quantified the fluorescence intensity of anti-RFP-Alexa647 at the IS (30% of the cell volume) *versus* the entire T cell. We compared cells overexpressing either one of the Fwe isoforms with Fwe KO and WT CTLs (Fig. 4E). Starting from 100% at the time of contact, Fwe KO CTLs exhibited little loss of fluorescence from the IS area, whereas WT CTLs exhibited a steady decrease in the fraction of RFP fluorescence at the IS over the 15-min recording period. Fwe KO CTLs expressing mFwe2 presented a loss of fluorescence at the IS, similar to WT cells. KO CTLs expressing either mFwe1 or mFwe3 presented a lower reduction in anti-RFP fluorescence at the IS than that observed in either the WT control or the mFwe2 construct, but the reduction was significantly greater (p < 0.001 for mFwe1 and p = 0.047 for mFwe3) than that observed in the Fwe KO CTLs. Loss of anti-RFP fluorescence in Fwe KO CTLs expressing the mFwe4 construct did not differ from that in the Fwe KO CTL. This result indicates that deletion of the N-terminus results in loss of function, while the C-terminal deletion also reduces function but to a lesser degree.

The confocal images suggest that endocytosed Syb2 is trafficked together with Fwe. To confirm this observation, we performed high resolution SIM imaging in Fwe KO CTLs overexpressing mFwe2-mTFP and Syb2-mRFP (Fig. 4F). The cells were fixed immediately after 30 min contact with P815 target cells in presence of the anti-RFP-Alexa647 antibody. We found that the mFwe-mTFP signal partially colocalized with the endocytosed anti-RFP-positive compartments and vice versa. This result suggests a transient localization of mFwe2 on endocytosed Syb2 vesicles.

The integrity of the mouse Fwe constructs was demonstrated by western blot as all constructs showed the expected molecular mass (Fig. S4B). Furthermore, no significant differences in the expression levels of the constructs were detected, indicating that the endocytosis phenotype is not due to variable protein expression levels. The analysis was conducted by analyzing the mTFP fluorescence intensities of the expressed mouse Fwe constructs in all cells used for the endocytosis assay (Fig. S4C).

The position of the mTFP tag could affect the efficacy of the mFwe2 construct function. Therefore, we expressed mFwe2 tagged with mTFP at its N- or C-terminus. Figure S4D shows exemplary images from time series of the endocytosis assay taken with Fwe KO CTLs expressing either construct. The anti-RFP fluorescence at the IS over time was similar for both constructs (Fig. S4D, E). Thus, the position of the TFP tag did not affect the efficacy of rescue.

### Mouse Flower isoforms polarize to the plasma membrane with different efficacies upon stimulation

**Fig. 5:**
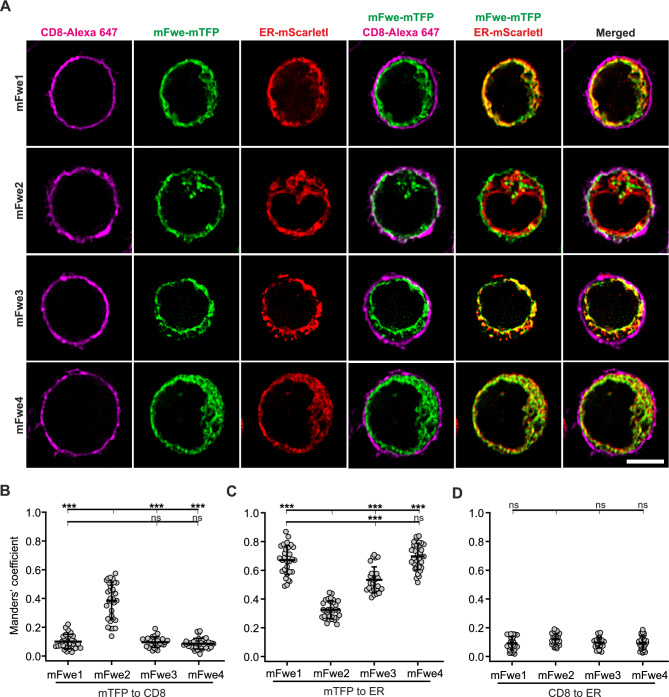
Mouse Flower 2 is localized on vesicular structures and the plasma membrane in bead stimulated CTLs. **A** Single plane SIM images of activated Fwe KO CTLs transfected with one of the four mouse Flower-mTFP isoforms and ER-mScarletI. After 14 h, cells were bead simulated for 1 h and stained with CD8-Alexa647 as a surface marker. The cells were plated on poly-L-ornithine coated coverslips, fixed with 4% PFA and imaged. Scale bar: 5 µm. **B**, **C** The Manders’ coefficient of colocalization between Flower isoforms and CD8 (**B**) and the ER marker (**C**). Means ± SD; n = 32, 29, 30, 25, respectively, N = 2, Kruskal–Wallis test followed by Dunn’s Multiple Comparison (*** p < 0.001, ns > 0.05). **D** Manders’ coefficient of colocalization of CD8 and ER markers (means ± SD; n = 32, 29, 30, 25, respectively, N = 2, Kruskal–Wallis test followed by Dunn’s Multiple Comparison, * p < 0.05, ** p < 0.01, *** p < 0.001, ns > 0.05)

We examined the cellular distribution of the four mouse Fwe-mTFP constructs expressed in Fwe KO CTLs. The PM was stained with an anti-CD8-Alexa647 antibody, and the ER was stained with an overexpressed ER-mScarletl construct (Fig. 5A). 12 to 14 h after transfection, the cells were stimulated with anti-CD3/anti-CD28 beads for one hour to mimic IS formation and to drive the Fwe protein into the membrane. Costaining of Fwe-mTFP with the plasma membrane (CD8) is visible for all mFwe isoforms. Scatter dot plots of Manders’ coefficients show that mFwe2 reached the plasma membrane to a higher degree (0.38 ± 0.02) than did the mFwe1, 3 or 4 constructs (Figs. 5B–D and S5A for better visualization). Mouse Fwe1 and 4 are predominantly found in the ER. Although mFwe1 and mFwe3 weakly colocalized with the plasma membrane, a partial rescue of Syb2 endocytosis was detected (Fig. 4E). Additionally, we could show that all functional mFwe isoforms (1 to 3) partially trafficked to the IS on common vesicular structures (Fig. S5B, C).

Since expression levels do not correlate with endocytosis rescue and there were significant differences in the cellular distributions of the Fwe constructs, we examined the oligomeric state of several selected constructs after expression in CTL. Figure S6 shows western blots of lysates (10 µg) of Fwe KO CTLs expressing mFwe1–4 prepared under non-denaturing conditions and probed with anti-DsRed antibody recognizing mTFP (native PAGE and western blotting; see Materials and Methods). The mFwe2 protein was present predominantly as a tetramer but was also clearly present as a dimer, with slightly higher-order oligomerization. Although mFwe1 and mFwe3 have different transfection efficiencies, as indicated by the weaker signal in the lane of the western blot (Fig. S6), these isoforms essentially appear as tetramers and hexamers. Mouse Fwe4 is present as tetramers, hexamers and higher-order oligomers. Together with our endocytosis rescue experiments, these results indicate that functional Fwe isoforms in mouse CTLs appear as dimers or tetramers.

### Mutation of the putative AP2 binding site YRWL at the N-terminus of mFwe2 and mFwe1 prevents rescue of Synaptobrevin2 endocytosis

We found that the deletion of the C-terminal end of mFwe (mFwe1 and 3) had a weaker effect on the ability to rescue Syb2 endocytosis than the N-terminus (mFwe4). A feature of both the N- and C-terminal exons is the putative AP2 binding motif YXXΦ [23], which could support an interaction with clathrin in endocytosis. Mutation of both AP2 binding motifs prevents Syb2-mRFP endocytosis [2, 15]. Our objective was to determine the individual contribution of each AP2 site to the protein's function and compare the mutation effects with those of the N- and C-terminal deletions.

**Fig. 6:**
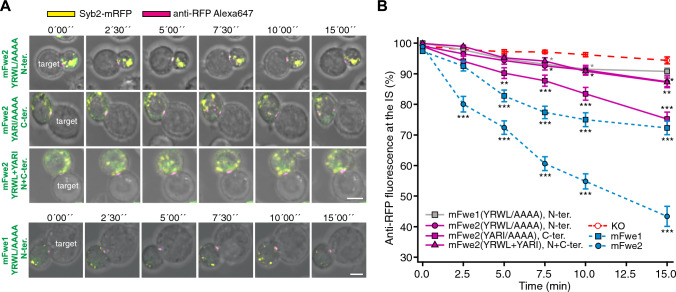
Mutation of the putative AP2 binding site YRWL at the N-terminus in mFwe2 and mFwe1 inhibits Synaptobrevin2 endocytosis. **A** Sequential images of Fwe KO CTL expressing Syb2-mRFP (yellow) and either mFwe2-mTFP mutants (N-terminus YRWL/AAAA, C-terminus YARI/AAAA and N- and C-terminus YRWL/AAAA and YARI/AAAA) or mFwe1(YRWL/AAAA)-mTFP. CTLs are conjugated with P815 target cells in the presence of anti-RFP-Alexa647 antibody. Scale bar: 5 μm. **B** Quantitative analysis of the distribution of anti-RFP-Alexa647 at the immunological synapse (IS) of Fwe KO CTLs expressing mFwe1 and mFwe2 mutants as described in **A** compared with that of Fwe KO CTL and Fwe KO CTL expressing mFwe1 and mFwe2 isoforms. Time zero was defined as the appearance of the first anti-RFP fluorescence at the IS. (Data given as means ± SEM; KO n = 19 (identical to Fig. 4E), N = 4; mFwe1 n = 21, mFwe2 n = 16, mFwe1(YRWL/AAAA)/N-terminus n = 22, mFwe2(YRWL/AAAA)/N-terminus n = 20, mFwe2(YARI/AAAA)/C-terminus n = 22, mFwe2(YRWL/YARI)/N- and C-termini n = 21, N = 3; Kruskal–Wallis test followed by Dunn’s Multiple Comparison *versus* KO control (* p < 0.05, ** p < 0.01, *** p < 0.001, ns > 0.05)

We generated constructs in which an N-terminal YRWL^26−29^ to AAAA, a C-terminal YARI^148−151^ to AAAA mutation, as well as a construct in which both sequences were mutated to AAAA in mFwe2. Western blots prepared from a lysate of Fwe KO CTL expressing these constructs ran at the expected molecular mass (Fig. S7A, left). The targeted mutation of YRWL to AAAA did not alter the oligomerization state of mFwe2, as shown by the native western blot in Fig. S7C. We overexpressed them in Fwe KO CTLs and conducted our Syb2 endocytosis assay. The expression levels of these constructs measured via the mTFP fluorescence intensities of the cells were similar, with the exception of the mFwe2 (YARI/AAAA) mutant construct, which was expressed at a greater level (Fig. S7A, right). The loss of anti-RFP-Alexa647 fluorescence at the IS is shown as time-lapse live snapshots over 15 min in Fig. 6A and is quantified in Fig. 6B. For comparison, mouse Fwe KO cells and Fwe KO CTLs expressing the mFwe1- and mFwe2-mTFP constructs, are shown as well. Mutation of the N-terminal AP2 site, or both AP2 sites, blocked the ability of mFwe2 to rescue endocytosis of Syb2-mRFP. Mutation of the C-terminal site reduced rescue, but this mutant performed significantly better than the N-terminal mutant compared with the KO control. The results for both single mutants were verified by SIM (Fig. S8 A, B). Additionally, this high-resolution imaging revealed that none of the AP2 mutant constructs accumulated at the IS, suggesting that they are not retained within the plasma membrane. This finding indicates that while the C-terminal AP2 site facilitates endocytosis, the N-terminal site is essential.

To prove that it is in fact the N-terminal AP2 site that confers its function to the mFwe isoforms lacking the C-terminus, we mutated YRWL to AAAA at the N-terminal AP2 site in mFwe1 (Fig. 6A). After expression in Fwe KO CTLs, this mutation also resulted in a strong reduction of Syb2 endocytosis rescue (Fig. 6B). The mFwe1 constructs ran at the expected molecular mass in western blots and the fluorescence levels in CTL were similar, ruling out differences in protein expression (Fig. S7B). These results show that the mutation of the N-terminal AP2 site of Fwe is sufficient to prevent rapid recycling of Syb2 mRFP in mouse CTLs.

### The human isoforms Fwe2 and Fwe4 and the *Drosophila* isoform dUbi rescue Syb2 endocytosis in Flower KO murine CTLs

**Fig. 7:**
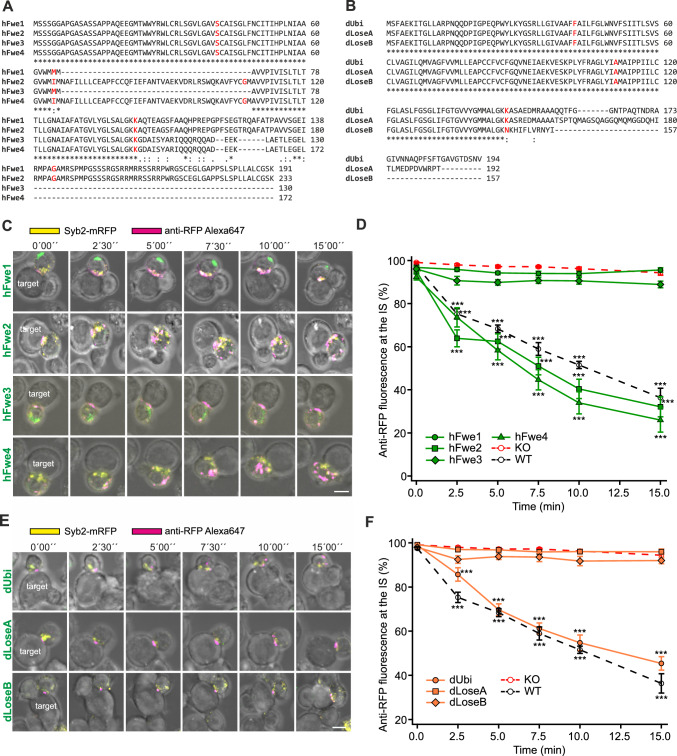
The human isoforms Fwe2 and Fwe4 and the *Drosophila* isoform dUbi rescue endocytosis in mouse Fwe KO CTLs. **A** Sequence alignment of human Flower isoforms hFwe1, hFwe2, hFwe3 and hFwe4 using Clustal Omega. * Indicates conserved amino acids. The exon/intron borders are shown in red. **B** Sequence alignment of *Drosophila* Flower isoforms dUbi, dLoseA and dLoseB using Clustal Omega. * Indicates conserved amino acids. The exon/intron borders are shown in red. **C** Time-lapse images of Syb2-mRFP fluorescence (yellow) of Fwe KO CTLs expressing each of the four hFwe-mTFP constructs (green) described in **A** conjugated to P815 target cells in the presence of anti-RFP-Alexa647 antibody (magenta). Scale bar: 5 μm. **D** Quantitative analysis of endocytosed Syb2-mRFP by anti-RFP-Alexa647 fluorescence at the immunological synapse (IS) in Fwe KO mCTLs expressing hFwe1-mTFP, hFwe2-mTFP, hFwe3-mTFP and hFwe4-mTFP constructs shown in **C** in comparison to those in WT and Flower KO mouse CTL. Time zero is defined as the appearance of the first endocytic signal at the IS. Data given as mean ± SEM; Kruskal–Wallis One-way Analysis of Variance on Ranks followed by multiple comparison (Dunn’s) was done against Fwe KO as control (KO n = 19, N = 4; WT n = 16 (identical to Fig. 4E), hFwe1 n = 11, hFwe2 n = 11, hFwe3 n = 11, hFwe4 n = 11, N = 3; *p < 0.05, **p < 0.01, *** p < 0.001). **E** Time-lapse images of Fwe KO mCTLs transfected with Syb2-mRFP (yellow) and each of the three *Drosophila* Fwe-mTFP isoform constructs (green) described in **B**. CTLs are conjugated to P815 target cells with anti-RFP-Alexa647 antibody (magenta) in the medium. Scale bar: 5 μm. **F** Quantitative analysis of endocytosed Syb2-mRFP fluorescence by anti-RFP-Alexa647 fluorescence at the immunological synapse (IS) in Fwe KO mCTL expressing dUbi-mTFP, dLoseA-mTFP or dLoseB-mTFP constructs, as shown in **E**, in comparison to WT and Fwe KO mCTL. Time zero was defined as the appearance of the first anti-RFP-Alexa647 signal at the IS. Data given as mean ± SEM; Kruskal–Wallis One-way Analysis of Variance on Ranks followed by multiple comparison (Dunn’s) was done against Fwe KO as control (KO n = 19, N = 4; WT n = 16 (identical to Fig. 4E), dUbi n = 17, dLoseA n = 16, dLoseB n = 14, N = 3; *p < 0.05, **p < 0.01, *** p < 0.001)

Human Fwe gene generates four splice variants, hFwe1-4 (Fig. 7A). Interestingly, two of these isoforms lack a central exon and show variability in their C-termini, allowing a more in-depth analysis of Fwe domains. In detail, hFwe1 and 2 differ from hFwe3 and 4 by their exons 5 and 6 at the C-terminus. hFwe1 is identical to the full-length hFwe2 with 233 amino acids (aa), but with a deletion of 42 aa beginning at aa 67 by exon skipping and additionally with a methionine at position 65. hFwe3 and hFwe4 have identical aa sequences, with the exception of the deletion mentioned above and the M65I aa exchange in hFwe4. All four human constructs contain the N-terminal YWRL site, and hFwe3 and 4 have the YARI sequence at the C-terminal site as well. In *Drosophila*, there are three splice variants (Fig. 7B), a ubiquitous form, dUbi, and two additional variants, dLoseA and dLoseB, which have been reported to generate “loser” phenotypes that tend to undergo apoptosis when competing with other cells [6]. The *Drosophila* isoforms differ among each other only in their C-termini starting from aa 147.

The human and *Drosophila* splice variants were expressed in mouse Fwe KO CTLs as described previously to measure their ability to rescue Syb2 endocytosis (Fig. 7C–F). A rescue, equivalent to that of the WT constructs, was achieved with both hFwe2 and hFwe4 constructs while the hFwe1 and hFwe3 constructs did not rescue Syb2-mRFP endocytosis (Fig. 7C, D). Thus, deletions of exon 3 in human isoforms prevent rescue while C-terminal deletions have little effect. Rescue with the dUbi construct exhibited Syb2-mRFP endocytosis like that of WT CTLs, whereas both the dLoseA and dLoseB constructs failed to promote endocytosis of Syb2-mRFP (Fig. 7E, F). The above results indicate that the isoforms hFwe2 and 4 as well as dUbi are capable of supporting Syb2 endocytosis in mouse Fwe KO CTLs.

We confirmed the molecular sizes of all the constructs and the similarity of fluorescence intensities in mouse CTLs after transfection (Fig. S9A, B). Western blot analysis from native PAGE hFwe4 exhibits dimerization and tetramerization while hFwe3 runs at higher molecular mass (Fig. S9C). The dUbi construct appeared predominantly as a dimer with a weak band at the tetramer level (Fig. S9D).

### Point mutations at the highly conserved tyrosine, Y104A in mFwe2 and Y109A in the *Drosophila* Ubi isoform, inhibit Synaptobrevin2 endocytosis

**Fig. 8:**
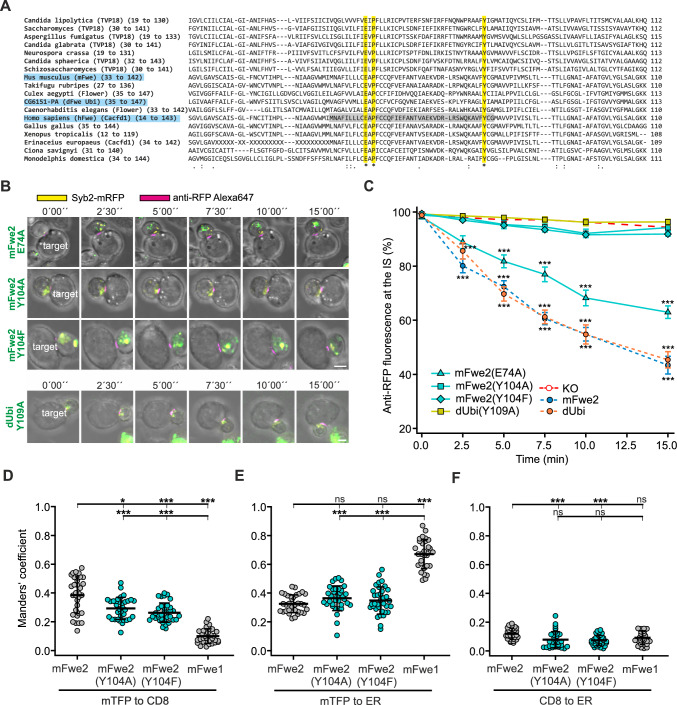
Point mutations in the highly conserved tyrosine residue Y104A in mFwe2 and Y109A in the *Drosophila* Ubi isoform block rescue of Synaptobrevin2 endocytosis. **A** Alignment of selected Cg6151-P-containing proteins in Clustal Omega format. The proteins include the TVP18 Golgi membrane proteins and Flower proteins from various species. The highly conserved glutamate (E), proline (P) and tyrosine (Y) residues are highlighted in yellow and marked with an *. The mouse, human and *Drosophila* sequences are highlighted in blue. The human Fwe equivalent of mouse exon 3 is shown in gray. **B** Images of Fwe KO CTLs expressing Syb2-mRFP (yellow) and one of the four mutated constructs, mFwe2(E74A)-mTFP, mFwe2(Y104A)-mTFP, mFwe2(Y104F)-mTFP or dUbi(Y109A)-mTFP, were acquired over 15 min. The transfected Fwe KO CTLs were conjugated to P815 target cells in the presence of anti-RFP-Alexa647 antibody (magenta). The first five frames were acquired at 2.5 min intervals, while the last image was acquired at 15 min. Scale bar: 5 μm. **C** Quantitative analysis of Syb2-mRFP endocytosis in Fwe KO mCTL expressing mFwe2 (E74A)-mTFP, mFwe2(Y104A)-mTFP, mFwe2(Y104F)-mTFP and dUbi(Y109A)-mTFP. Also shown are the traces for mFwe2-mTFP and dUbi-mTFP expressed in Fwe KO CTL and for the Fwe KO mCTL. The percentage of anti-RFP fluorescence at the IS is shown *versus* time. Time zero is defined as the appearance of the first anti-RFP-Alexa647 signal at the immunological synapse. The results are presented as the mean ± SEM; Kruskal–Wallis One-way Analysis of Variance on Ranks followed by multiple comparison (Dunn’s) was done against Fwe KO as control (KO n = 19 (identical to Fig. 4E), N = 4; WT n = 16, mFwe2(E74A)-mTFP n = 20, mFwe2(Y104A)-mTFP n = 24, mFwe2(Y104F)-mTFP n = 25, dUbi(Y109A)-mTFP n = 18, N = 3; *p < 0.05, **p < 0.01, ***p < 0.001). **D** Colocalization of mFwe2-mTFP, mFwe1-mTFP, mFwe2(Y104A)-mTFP and mFwe2 (Y104F)-mTFP with the plasma membrane marker CD8 as shown in Fig. 5A and B. The Manders’ coefficient (mTFP to CD8, means ± SD; n = 32, 34, 35, 29, respectively, N = 2, Kruskal–Wallis test followed by Dunn’s Multiple Comparison, *** p < 0.001, ns > 0.05). **E** Colocalization of Flower isoforms, as described above, and the ER marker as shown in Fig. 5A and C. Manders’ coefficient (means ± SD; n = 32, 34, 35, 29, respectively, N = 2, Kruskal–Wallis test followed by Dunn’s Multiple Comparison, *** p < 0.001, ns > 0.05). **F** Colocalization of Flower isoforms, as described above, with CD8 and ER as shown in Fig. 5A and D. Manders’ coefficient (means ± SD; n = 32, 34, 35, 29, respectively, N = 2, Kruskal–Wallis test followed by Dunn’s Multiple Comparison, * p < 0.05, ** p < 0.01, *** p < 0.001, ns > 0.05)

The results of the rescue experiments with the human isoforms indicate that the equivalent sequence found in mouse exon 3 is critical, as shown in hFwe1 and 3 (Fig. 7D). Deletion of exon 3 of mFwe1 (mFwe1(Δex3)) resulted in a complete loss of endocytic function (Fig. S10A–C). Fwe is included in a family of small proteins (less than 200 residues) that contain a Cg6151-P domain, which is conserved from fungi to humans. The alignment of various proteins from this Cg6151-P family in 16 different species, including mouse, human and *Drosophila* Fwe, is shown in Fig. 8A [24, 25]. Within the aligned sequences corresponding to the human exon 3 (highlighted in gray), only a glutamate (E74 in mouse), a proline (P76 in mouse) and a tyrosine (Y104 in mouse) are conserved (Fig. 8A, highlighted in yellow and marked *). Additionally, two (E, P) of these three amino acids are comprised in a sequence that has been identified as the selectivity filter in some TRP channels in *Drosophila* [1]. These authors exchanged the glutamate (E79) with a Q in *Drosophila* WT-Flower-PB and reported a loss of endocytosis in *Drosophila* salivary gland cells. We targeted mutations in the full-length mFwe2 at the E74 and Y104 residues and in the equivalent Y109 in dUbi and confirmed the integrity of all the constructs (Figs. S11A and S9E, respectively). Syb2 endocytosis analysis was done as described above in Fwe KO CTLs (Fig. 8B, C). The change in anti-RFP fluorescence at the IS in Fwe KO cells expressing the mFwe2 (E74A) mutation was significantly higher than that in the Fwe KO CTL (p < 0.001) but lower than that in the mFwe2 expressing cells (p = 0.712, Kruskal–Wallis test followed by Dunn’s Multiple Comparison *versus* control). Mutation of Y104A in mFwe2 resulted in a dramatic loss of Syb2 endocytosis, as did mutation of Y104 to F (91.88% ± 0.76 at 15 min). High-resolution imaging of this construct confirmed this observation and revealed the localization of the construct at the IS (Fig. S8C). The equivalent Y109A mutation in *Drosophila* Fwe isoform dUbi prevented rescue as well (Fig. 8B, C). These point mutations in mFwe2 and dUbi had no influence on their expression levels, as the cells expressing the constructs displayed comparable mTFP fluorescence intensities (Figs. S11B and S9E, respectively). Manders’ coefficient analysis showed that the mFwe constructs with point mutations (Y104A or Y104F) reach the plasma membrane to a lesser degree than WT mFwe2 but to a greater degree than mFwe1, a mouse variant that partially rescues Syb2 endocytosis (Fig. 8D). The localization of the mutants in the ER was comparable to that of mFwe2 but significantly lower than that of mFwe1 (Fig. 8E).

Western blots of native PAGE indicate that the point mutations shift the expressed proteins to higher order oligomers, with the strongest effects seen in the Y104 mutations (Fig. S11C) while mutation of the E residue produces modest oligomerization. A higher oligomerization state was also observed in the equivalent in *Drosophila* dUbi (Y109A) construct (Fig. S9D). Mutation of this tyrosine results in loss-of-function. Therefore, it is not because these constructs do not reach the plasma membrane that they are not able to rescue endocytosis. Instead, the loss of function is probably due to protein oligomerization.

### Flower isoforms and mutants can be classified into rescuer, partial and non-rescuer

**Fig. 9:**
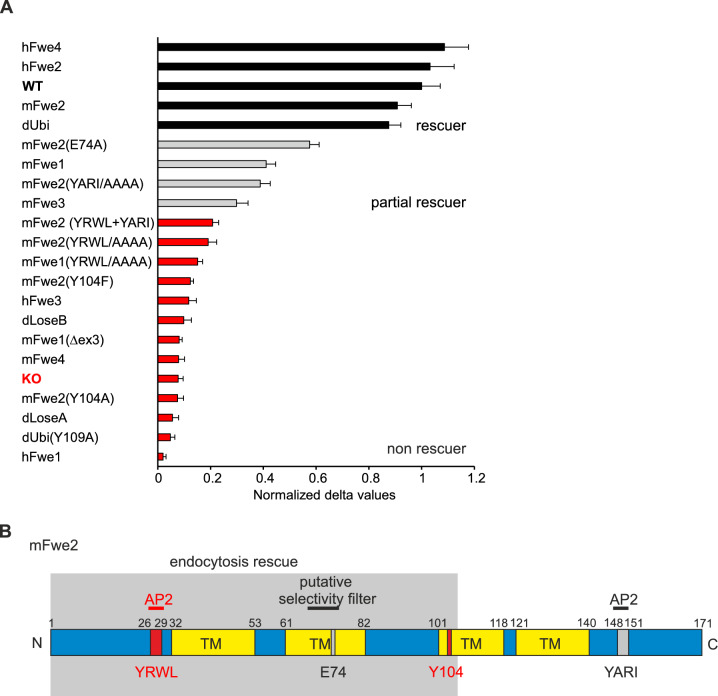
Classification of the analyzed Flower isoforms and mutants into rescuer, partial- and non-rescuer. **A** Bar graph with all analyzed Flower isoforms and mutants sorted by the normalized delta values calculated from time points 0 min and 15 min. Rescuers show > 80% rescue, partial rescuers 30–60% and non-rescuers < 30% endocytosis rescue. Data given as mean ± SEM. **B** The secondary structure of the full-length mouse Fwe2 protein with transmembrane domains (TMs) predicted by the CCTOP prediction tool, similar to the previously postulated topology (Yao et al., 2017, Chang et al., 2018). The mutations and the putative selectivity pore are drawn in red and gray, and the minimal region necessary for endocytosis rescue is drawn in the gray box according to (**A**)

Following the analysis of all our rescue experiments, we categorized different Fwe isoforms and mutants into three groups: rescuer, partial rescuer, and non-rescuer (Fig. 9A). The mean rescue reached at 15 min was normalized to the WT result (mean ± SEM). The “rescuers” mFwe2, hFwe2 and 4 and dUbi isoforms restored Syb2 endocytosis near the WT level. The next group, “partial rescuers”, performed significantly better than the KO rescue but significantly worse than WT cells and contain the mFwe2(E74A), the mFwe1, mFwe2(YARI/AAAA), and the mFwe3 constructs. In the last group, the “non-rescuers” are the mFwe constructs with N-terminal mutations of the putative AP2 site and the AP2 double mutant. This group includes a number of constructs with large deletions, such as mFwe1(Δex3), mFwe4, hFwe1 and hFwe3; the *Drosophila* isoforms dLoseA and dLoseB; and functional constructs with critical point mutations such as mFwe2(Y104A), mFwe2(Y104F) and dUbi(Y109A).

This led us to identify the minimal Fwe domain and critical amino acids required for CG endocytosis in CTL. Figure 9B shows the results in terms of mouse Fwe structure and the location of the mutations carried out. Fwe is shown in 2D with four putative transmembrane domains (TM) predicted by CCTOP prediction tool [26] postulating an intracellular localization of the N and C-termini, similar to a previously postulated topology [27, 28]. The AP2 sites and point mutations at the conserved glutamate and tyrosine residues are indicated, as is the position of the putative selectivity filter. For rescue of endocytosis, the N-terminal (aa 1 to 106) sequence is required. The amino acids 104 to 106 appear to be important for correct quaternary structure formation and stabilization of the Fwe protein at the plasma membrane, as shown by the lack of rescue with mFwe1(Δex3) and mFwe (Y104A or F).

## Discussion

At the immunological synapse (IS), Fwe is required for efficient endocytosis of Syb2, which allows regeneration of CG and is necessary for serial killing [2]. The recycling of Syb2 at the immunological synapse is a clathrin- and dynamin-dependent process [13]. Fwe also supports clathrin-mediated endocytosis at the neuromuscular junction in *Drosophila* [1]. Our results indicate that the endocytosis of transferrin receptor 1 (TfR1), which is a classical marker for clathrin-mediated endocytosis [29], proceeds normally in Fwe KO CTLs (Fig. 1A). Loss of Fwe did not phenocopy clathrin inhibition by pitstop2 [16]. Thus, clathrin-mediated endocytosis per se is not blocked by the loss of Fwe in CTLs. Similarly, LAMP1 recycling in unstimulated effector cells is not affected by Fwe KO [2]. In contrary, in target cell stimulated CTLs, LAMP1 endocytosis at the IS was reduced by the loss of Fwe (Fig. 1C, D). This change is modest relative to the reduction in endocytosis observed for Syb2, which is nearly obliterated in Fwe KO CTLs. Compared with LAMP1, Syb2 is a more specific marker for CG membrane [11]. Trafficking of LAMP1-positive lysosomal organelles is ongoing and much larger in volume than that of CG fusion at the IS, diluting the effect of Fwe KO on LAMP1 endocytosis. These results indicate a specificity for Fwe to mediate CG membrane uptake upon stimulation at the IS. The question is what provides this specificity. Endogenous mFwe2 is present only at low levels in the plasma membrane of mouse CTLs (Fig. 2F), suggesting why it is not involved in general clathrin-mediated endocytosis. The main reservoir of Fwe is small clear vesicles (Fig. 2C, D), which we identified for the first time in CTL. These are actively delivered to the IS following CTL stimulation by contact with target cells (Fig. 2A, B). This increase in the density of Fwe in the plasma membrane at the IS appears necessary for targeting Syb2 endocytosis (Fig. 2F). Our results indicate that Fwe is not required for endocytosis per se but is delivered to the IS, where it likely forms a recruitment platform for clathrin-mediated endocytosis, directing the CG membrane with its complement of Syb2, LAMP1 and possibly other CG proteins into the clathrin-mediated endocytosis pathway.

In mouse CTL, four main Fwe isoforms are expressed (Fig. 3C, E). mFwe2 is the full-length protein that fully rescues endocytosis in Fwe KO cells [2]. The short mFwe1 and mFwe3 isoforms, characterized by missing exons 4–5 and a variable amino acid sequence of 22 aa at the C-terminal end, respectively, partially rescue Syb2 endocytosis in Fwe KO mCTLs (Figs. 4D–E, 9A), indicating that the C-term is dispensable. mFwe4 did not rescue Syb2 endocytosis in Fwe KO CTLs (Fig. 4D–E), suggesting that the N-terminus is required. We tested this hypothesis by performing site directed mutations of putative AP2 binding sites characterized by two tyrosine-based sorting signal sequences YXXФ [14], located either at the N- or C-terminal part of the protein. Fwe interacts with AP2 in CTL, and mutation of both AP2 sites together disrupts this interaction [2, 15]. Targeted mutation of the YRWL motif located at the N-terminal of mFwe2 confirmed its importance for Syb2 endocytosis. Mutation of the second putative AP2 binding motif, YARI, in the C-terminal region of mFwe2 still allowed partial rescue of endocytosis (37.13 ± 3.8% of control), supporting the notion that the Fwe C-terminus is not critical for Syb2 endocytosis function (Figs. 6A, B and 9A). The N-terminal potential AP2 sequence, YRWL, is involved in this recognition step, though its function as an AP2 binding sequence has not been proven. In *Drosophila,* an N-terminal sequence (28–33, LKYGSR) was identified as a potential PIP2 binding site, and the positively charged residues K29 and R33 were mutated to alanine, resulting in loss of function. In mouse and human Fwe, this corresponds to the sequence YRWLCR, which also contains two positively charged residues, including the R of the YRWL sequence. Although this mutation affects PIP2 binding in Drosophila, which is reported to modulate the calcium conductance of Fwe, the calcium conductance of Fwe does not appear to be involved in the regulation of clathrin-mediated endocytosis [28, 30].

Additional explanations for loss of function are mis-localization, mis-folding or protein aggregation. mFwe1 and 3, which induced a partial endocytosis rescue, had a reduced plasma membrane localization. Although these isoforms are thought to have only two transmembrane domains [4, 28], they also appear mainly as dimers and tetramers, similar to fully functional mFwe2 (Fig. S6). In contrary, mFwe4 is retained in the ER and tends to aggregate with higher-order oligomerization (Figs. S6 and 5C). This might suggest that Fwe proteins function as dimers and tetramers.

In addition to the mouse Fwe protein, the function of human and *Drosophila* Fwe proteins have been studied [1, 3, 28, 30]. They exist in many isoforms in which various parts of the protein are missing, allowing us to refine the study of Fwe functional domain for endocytosis. Therefore, we tested the human isoforms Fwe1–4 and the *Drosophila* isoforms dUbi, dLoseA and dLoseB. The human isoforms differ mainly at their C-termini and at a 42 aa deletion of human Fwe exon 3 (aa 66–107), whereas the *Drosophila* isoforms show 100% sequence homology from aa 1 to 146 and differ only in length and in their C-termini (Fig. 7B). The human isoform Fwe4 is highly homologous (158/170 identities, 93%) to mFwe2 [31], while hFwe2 shows amino acid sequence changes starting at aa 143 (Fig. 7A). Both isoforms support Syb2 uptake and are "rescuers" comparable to mFwe2 (> 91.7%) rescue (Fig. 9A). This confirms the modest influence of the AP2 binding site (YARI motif) at the C-terminus (absent in hFwe2).

The human isoforms hFwe1 and hFwe3 differ from hFwe2 and hFwe4 in exons 5 and 6 and in the absence of exon 3 (Fig. 7A). Both hFwe1 and hFwe3 are "non-rescuers" (< 30% rescue, Fig. 9A), confirming the importance of this sequence. Greater aggregation (Fig. S9C) is consistent with an important role in the formation of the tertiary or quaternary protein structure for exon 3. Its absence may alter the orientation of hFwe1 and hFwe3 in the plasma membrane, particularly the orientation of the N-terminus, as described in Madan et al. [3]. In agreement with this interpretation, it was recently shown that hFwe3 is predominantly sequestered in the ER [15]. Deletion of the sequence equivalent to human exon 3 in mFwe1 blocked the rescue of Syb2 endocytosis as well (Fig. S10).

Although we did not expect that the *Drosophila* isoforms would be properly processed and folded in mammalian cells, because of their low amino acid identity (36%) with mFwe2, the dUbi isoform supported Syb2 endocytosis in murine CTL. In contrast, dLoseA and dLoseB did not support Syb2 endocytosis in mCTL. In *Drosophila*, mutation of glutamate (E79Q), which is expected to interact with cations and is integral to the cation selectivity filter, reduces the increase in calcium associated with the expression of Fwe to KO levels [1].

Our experiments showed the importance of exon 3 for the function of Fwe in endocytosis, in which three amino acids (E, P, and Y) are highly conserved among species (Fig. 8A). This conserved region of the Fwe protein contains a putative calcium selectivity filter [1] consisting of a 9 aa motif (aa 68 to 76, Fig. 9B) localized in TMD2. Mutations in the glutamate residues of the putative filter in mFwe2 confirm the importance of this sequence, but it is not clear that Fwe actually functions as a calcium channel [27, 32]. In CTL, the mutation of the conserved glutamate in mFwe2 (E74A) led to a modest reduction in Syb2 endocytosis efficacy (Fig. 9A, partial rescuer, 57.5% of control). Thus, Syb2 endocytosis appears to not require Fwe calcium channel function. This finding is consistent with recent results showing that in *Drosophila*, activity-dependent bulk endocytosis requires a Fwe-dependent calcium increase, but Fwe support of clathrin-mediated endocytosis resulting from moderate activity at the neuromuscular junction is independent of its calcium channel function [28]. In contrast, mutation of the highly conserved tyrosine (Y104) in mFwe2 and its corresponding position Y109 in *Drosophila* led to a complete loss of function. This tyrosine is likely localized at the transition or inside the third TMD (Fig. 9B). Y104A and Y104F mutations in mFwe2 resulted in lower PM localization, but these constructs reached the PM significantly better than did mFwe1, which still exhibited partial rescue (Figs. 8D and 9A). Mutation of this Y residue resulted in a shift to higher-order multimers (Fig. S9D and Fig. S7C). Preponderance of higher-order oligomers in native gels does not rule out the presence of dimers or tetramers at the PM. In *Drosophila*, rescue was achieved with expression levels near 4% of endogenous expression, indicating that only a fraction of the expressed protein is sufficient for function [28]. Since there is no evidence predicted by NetPhos-3.1 [33] that this tyrosine is phosphorylated, it is likely required for correct anchoring in the plasma membrane and/or interaction between subunits to stabilize quaternary structure in mFwe2 and dUbi.

We categorized the different isoforms and mutants as rescuers (80–100%), partial (30–80%) and non-rescuers (< 30%) based on their endocytosis rescue efficacy (Fig. 9A). Accordingly, the equivalent of exons 1, 2 and 3 of mFwe2 (N-terminus und TMD1 und 2) is sufficient for protein function in Syb2 (Fig. 9B). Full rescue is achieved by mFwe2, hFwe2 and 4, and dUbi. All other isoforms perform significantly worse in rescue of endocytosis of Syb2 in mCTL.

In addition to the importance of Fwe in endocytosis, Fwe influences cell fitness [3, 4, 6]. Expressed Fwe isoforms are fitness indicators that determine whether cells compete successfully (Win) or not (Lose) and play important roles in development and metastasis. This fitness code is consistent with the ability of human and *Drosophila* isoforms to rescue Syb2 endocytosis. However, there is a discrepancy in the mFwe isoforms. mFwe1 and 3 are Loser isoforms, and mFwe2 and mFwe4 are Winner isoforms [4]. Since mFwe4 is a Win isoform that does not support endocytosis and mFwe1 is a Lose isoform that supports endocytosis, the fitness code does not appear to be related to Fwe function in endocytosis.

In addition to the N-terminal sequence, we identified other regions in the Fwe protein that are important for endocytic function. Exon 3, particularly the tyrosine residue, is important for correct localization in the plasma membrane and quaternary structure formation, and its mutation results in complete loss of function. Finally, we identified the minimal protein domain required for Syb2 endocytosis rescue in mCTLs (Fig. 9B). The extent to which a direct interaction between Fwe and Syb2 plays a role, whether Fwe acts as a central hub for the endocytosis of other proteins, and which Ca^2+^ source is required to overcome the endocytosis defect in Fwe-deficient CTLs [2] requires further investigation.

## Supplementary Information

Below is the link to the electronic supplementary material.

Supplementary file1 (PDF 4538 KB)

## Data Availability

Data that support the findings of this study, as well as the plasmids generated in this study, are available from the corresponding author upon request.
