# Correction: Required minimal protein domain of flower for synaptobrevin2 endocytosis in cytotoxic T cells

**DOI:** 10.1007/s00018-025-05619-7

**Published:** 2025-03-13

**Authors:** Keerthana Ravichandran, Claudia Schirra, Katja Urbansky, Szu-Min Tu, Nadia Alawar, Stefanie Mannebach, Elmar Krause, David Stevens, C. Roy D. Lancaster, Veit Flockerzi, Jens Rettig, Hsin-Fang Chang, Ute Becherer

**Affiliations:** 1 Cellular Neurophysiology, Center for Integrative Physiology and Molecular Medicine (CIPMM), Saarland University, 66421 Homburg, Germany; 2 Department of Structural Biology, Center of Human and Molecular Biology (ZHMB), Faculty of Medicine Building 60, Saarland University, 66421 Homburg, Germany; 3 Experimental and Clinical Pharmacology and Toxicology and Preclinical Center for Molecular Signaling, Saarland University, Homburg, Germany

**Correction: Cellular and Molecular Life Sciences (2024) 82:8** 10.1007/s00018-024-05528-1

In this article Fig. 2 appeared incorrectly and have now been corrected in the original publication. For completeness and transparency, the old incorrect and the corrected versions are displayed below.

Incorrect version:
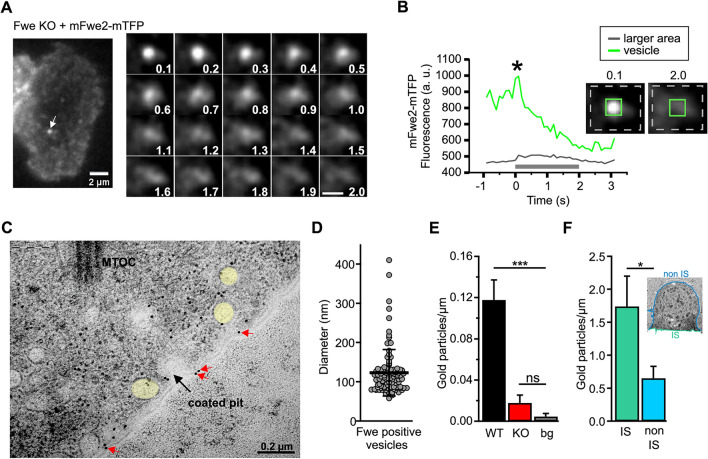


Corrected version:

**Fig. 2:**
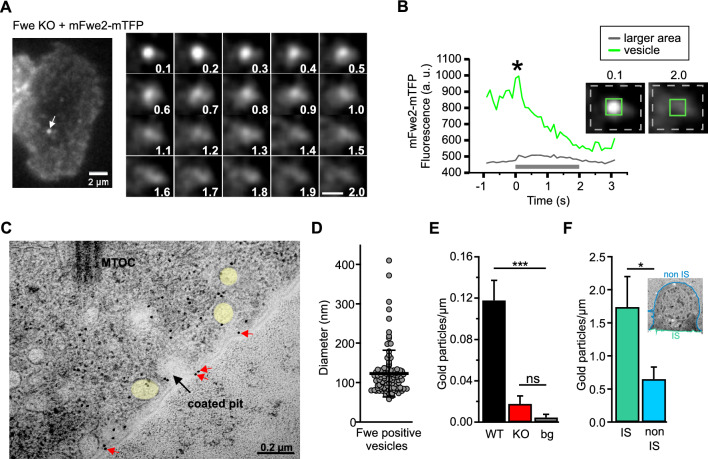
Mouse Flower 2 is localized on small vesicles that fuse with the plasma membrane upon T cell stimulation. **A** Live cell TIRFM imaging of mFwe2-mTFP expressing Flower KO CTL. Snapshot images of an exemplary cell and a Flower fusion event. Overall, 20 fusion events were measured in 11 cells expressing either mFwe2-mTFP or mScarletI-mFwe2-mTFP. The images were recorded at 10 Hz. Scale bar 2 µm and 1 µm, respectively. **B** Fluorescence intensity time course of the mFwe2-mTFP-positive vesicle shown in **A**. The green line shows the fluorescence intensity at the vesicle (insert with green square), whereas the gray line corresponds to the intensity of a larger membrane area (insert with stippled gray rectangle). Fusion occurs when the green line shows maximum fluorescence intensity (*). Its decay indicates dispersion of the mFwe2-mTFP in the plasma membrane following fusion of the vesicle. Accordingly, a slight increase in fluorescence is observed in the surrounding plasma membrane. The gray bar corresponds to the time of the snapshots shown in **A**. **C** A representative electron micrograph showing part of the immunological synapse of a CTL expressing mFwe2-mTFP. Post-embedding immunogold electron microscopy was done on activated mCTLs settled on anti-CD3ε coated sapphire discs. The protein was labeled with the primary antibody, anti-tRFP against mTFP, and with a secondary gold-conjugated antibody (10 nm). The microtubule organizing center (MTOC) is present, as are Fwe-positive vesicles (yellow). Flower was identified on the plasma membrane (red arrows) and on a coated pit (black arrow). Scale bar, 0.2 µm. **D** Quantitative analysis of the diameter of immunogold-labeled Flower positive vesicles as shown in **C**. Data given as mean ± SEM (N = 4, n_cells_ = 14, n_vesicles_ = 99). **E** Quantitative analysis of endogenously expressed Flower protein in WT and KO mCTLs localized on the plasma membrane (shown in **C**, red arrows). Post-embedding, immune electron microscopy was done with primary anti-Flower antibody and 10 nm secondary goat anti-rabbit gold antibody on ultrathin sections cut parallel to the sapphire disc. For background control (bg) sections were stained without primary antibody. Gold particles were counted per µm. Data given as mean ± SEM. Kruskal–Wallis One-way Analysis of Variance on Ranks followed by multiple comparison (Dunn’s) was done. (N = 3; WT n = 19, KO n = 18, bg n = 21; ***p < 0.001, not significant (ns)). **F** Quantitative analysis of overexpressed mFwe2-mTFP protein localized on the plasma membrane. Post-embedding immunogold electron microscopy was done as described in **C** with primary anti-Flower antibody and 10 nm secondary goat anti-rabbit gold antibody on ultrathin sections cut vertical to the sapphire disc. Inset shows a representative image of an activated CTL, settled on anti-CD3ε coated sapphire disc with marked immunological synapse (IS, green) and non IS regions (blue). Gold particles localized on the plasma membrane, as shown in **C**, were counted per µm (mean ± SEM (N = 3; non IS n = 18)). Data significance was analyzed by Mann–Whitney Rank Sum Test (*p < 0.05)

The original article has been corrected.

