# Supplementary material for: Required minimal protein domain of flower for synaptobrevin2 endocytosis in cytotoxic T cells
Source: Cell Mol Life Sci. 2024 Dec 18;82(1):8. doi: 10.1007/s00018-024-05528-1 (PMC11655906; doi:10.1007/s00018-024-05528-1)
Supplement: Supplementary file 1 — Supplementary file1 (PDF 4538 KB) [file 18_2024_5528_MOESM1_ESM.pdf]

Required Minimal Protein Domain of Flower for Synaptobrevin2 Endocytosis in Cytotoxic T Cells

Keerthana Ravichandran[1,4], Claudia Schirra[1,4], Katja Urbansky[2], Szu-Min Tu[1], Nadia Alawar[1], Stefanie Mannebach[3], Elmar Krause,[1] David Stevens[1], C. Roy D. Lancaster[2], Veit Flockerzi[3], Jens Rettig[1], Hsin-Fang Chang[1*] and Ute Becherer[1*]

Supplementary Figures

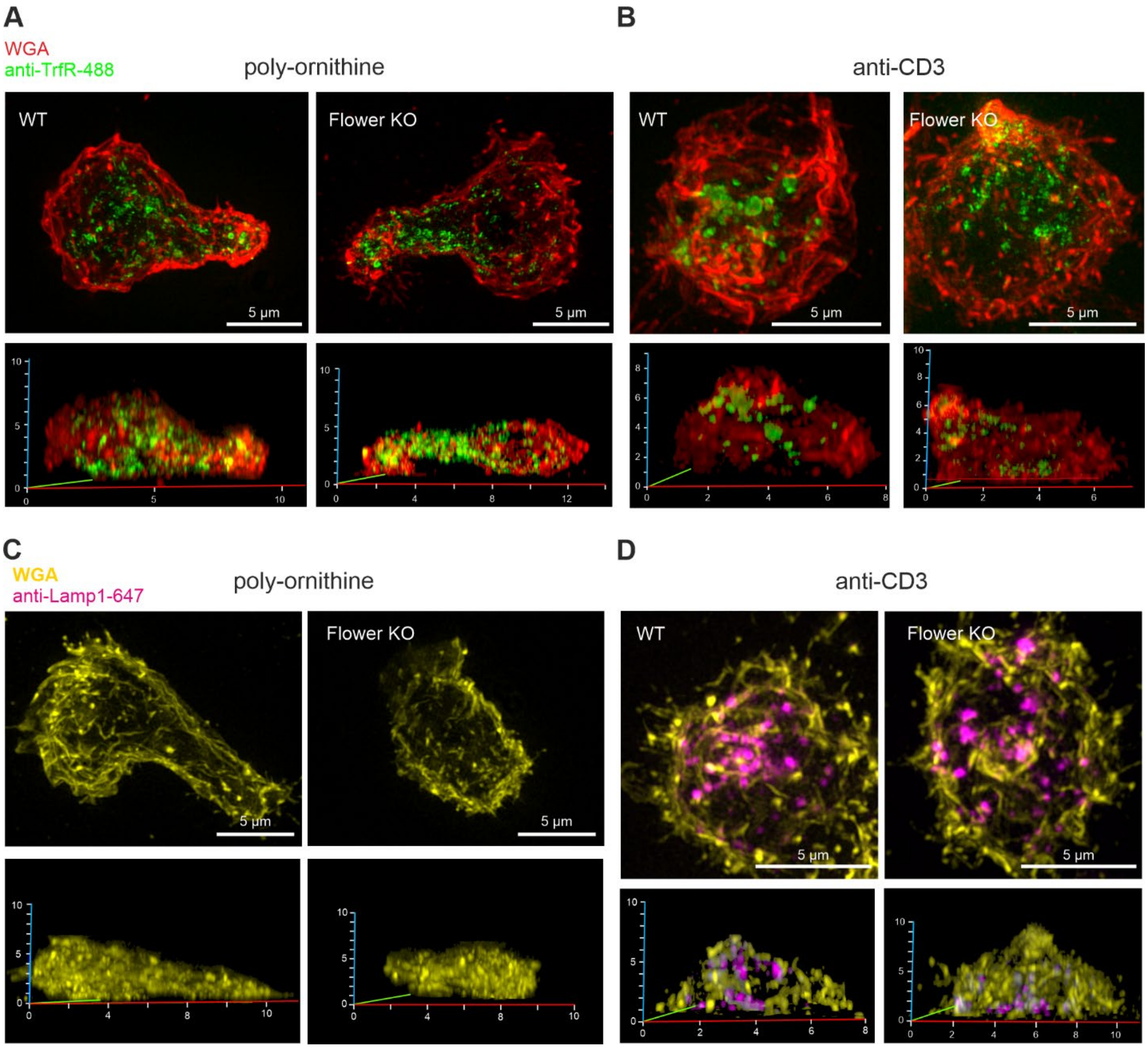

Supplementary Figure 1 (related to Fig. 1): Flower mediates LAMP1 endocytosis in a stimulation-dependent manner and does not contribute to the global endocytosis of TfR1. SIM images of WT and Flower KO CTLs plated on poly-L-ornithine (A and C) or anti-CD3ε antibody-coated coverslips for 40 min (B and D) to trigger synapse formation and fixed immediately thereafter. The cells were treated with the corresponding antibodies directly following their seeding on the coverslip. Presented are MIP images (top) and 3D images (bottom) **(A-B)** Anti-TfR1-Alexa488 antibody (green) was applied to label endocytic TfR1. The plasma membrane was labeled with WGA-Alexa594 (red). **(C-D)** Anti-LAMP1-Alexa647 antibody (magenta) was applied to label endocytic LAMP1. The plasma membrane was labeled with WGA-Alexa594 (yellow).

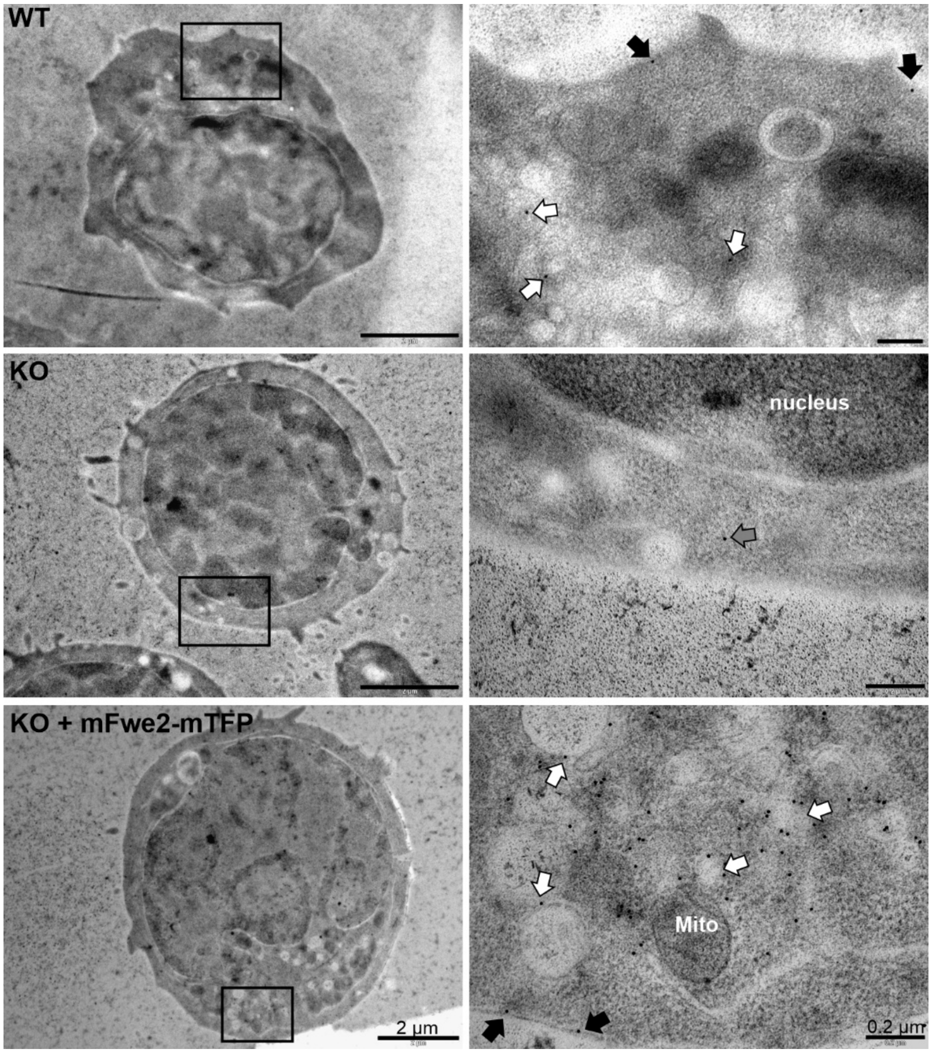

Supplementary Figure 2 (related to Fig. 2): Endogenous mFwe2 is localized on small vesicles and on the plasma membrane. Representative electron micrographs of CTLs from WT, Fwe KO and Fwe KO expressing mFwe2-mTFP. Post-embedding immunogold electron microscopy was performed on activated CTLs settled on anti-CD3ε-coated sapphire discs. The protein was labeled with primary anti-flower antibody and 10 nm secondary goat anti-rabbit gold antibody on ultrathin sections. The **left** images show the CTL marked with an ROI magnified in the **right** panel. **Top row:** The left image shows a mouse WT CTL. The enlarged image on the right shows immunogold labeled endogenous Fwe protein localized on the plasma membrane (black arrows) and on vesicular structures in the cytosol (white arrows). **Middle row:** Fwe KO CTL is shown on the left. The grey arrow marks an unspecifically localized gold particle in the cytoplasm in the enlarged area of the KO cell (right). **Bottom row:** KO CTL expressing mFwe2-mTFP (left). The magnified area of the cell shows immunogold particles localized on vesicular structures (white arrows) and on the plasma membrane (black arrow). Scale bars: 2 µm and 0.2 µm. Data were generated from two independent experiments (N=2, KO n=13, WT n=14, mFwe2-mTFP n=15)

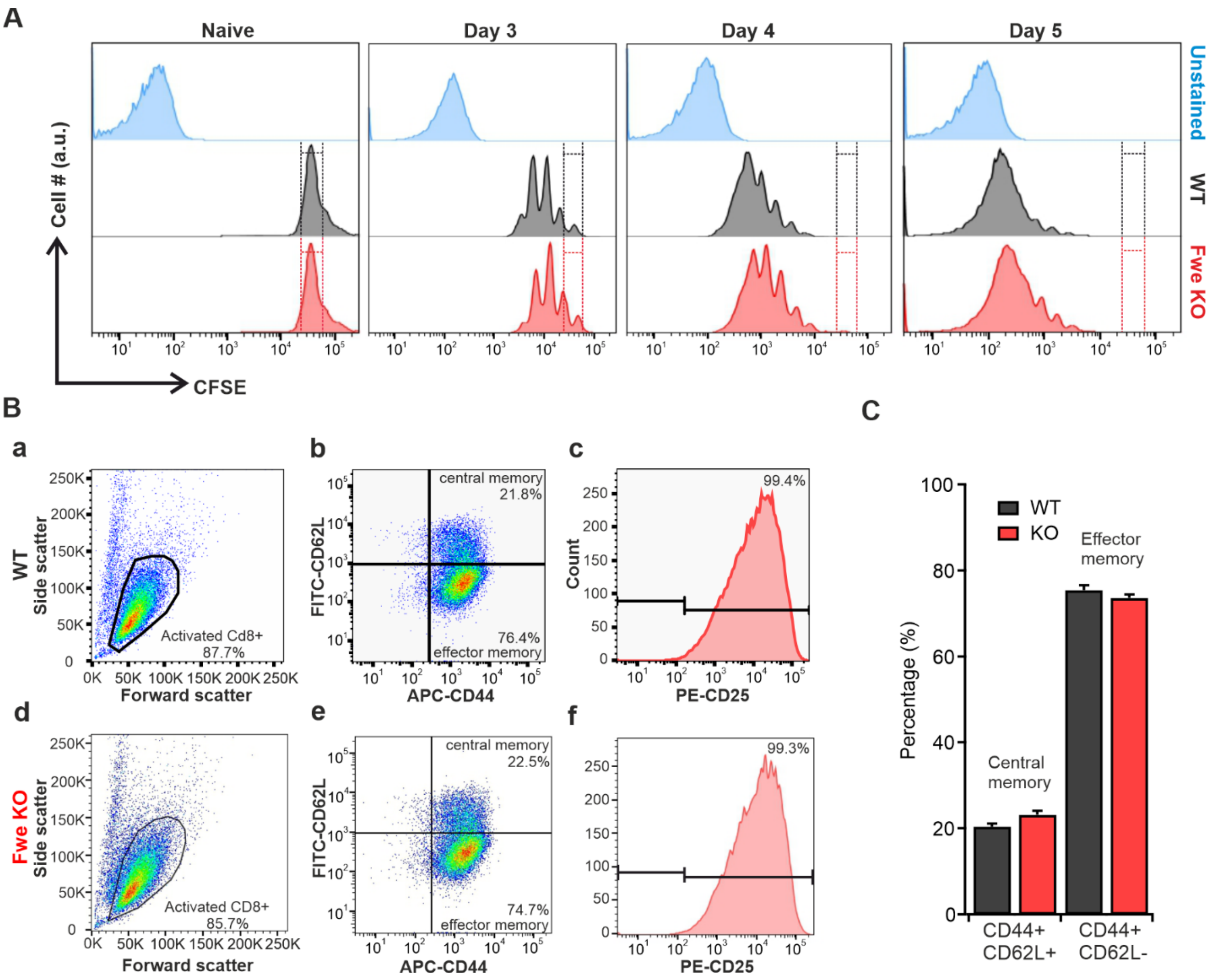

Supplementary Figure 3 (related to Fig. 3): Flower KO CTL show similar proliferation and activation state compared to WT cells. (A) Naive primary CD8$^+$ T cells from WT (black) and Fwe KO (red) were labelled with 5 µM of CFSE and cultured for 5 days using anti-CD3/CD28 dynabeads. Flow cytometric analysis was done on day 3, 4 and 5 to determine the intensity of CFSE staining using excitation of 488 nm. The histogram gating was done based on CD8$^+$ T cells of 85% to 90% live population. The different peaks in the histogram represent different cell generation stages. Gating in dotted lines represents undivided cell population. Unstained cells (blue) were used as baseline control for auto-fluorescence. **(B)** Flow cytometry analysis of day 5 activated CTL to study the subtype population using surface markers CD62L and CD44. CD8$^+$ WT CTL were activated for five days and stained using CD62L-FITC, CD44-APC and CD25-PE antibodies. Upper panel: (a) Healthy CTL were first selected based on forward and sideward scatter. (b) Those cells were plotted according to the surface markers CD62L and CD44. (c) In addition, the general activation state of cells was analyzed using the surface marker CD25. Lower panel: (d-f) same as described in (a-c) for Fwe KO cells. 74.7% were effector memory and 22.5% central memory cells. All gating was done in comparison to unstained control (not shown). **(C)** Quantification and average percentage of effector and central memory cells of WT and Fwe KO CTL from three independent experiments (N=3; values shown as mean ± SEM).

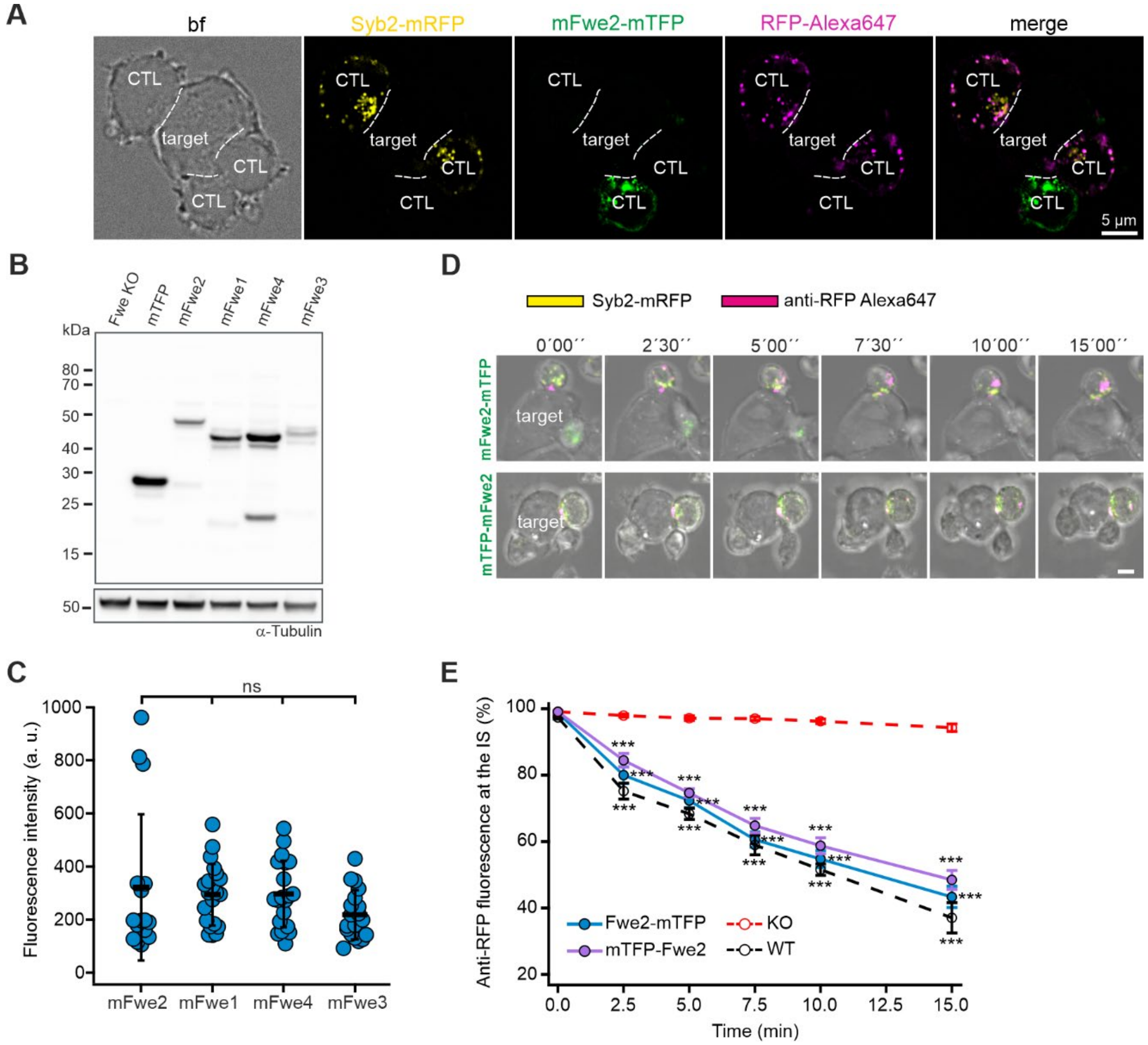

Supplementary Figure 4 (related to Fig. 4): Endocytosis rescue of mFwe2 is independent of mTFP-tag position. (A) Single plane SIM image of mouse KO CTLs electroporated with mFwe2-mTFP and Syb2-mRFP, treated with the anti-RFP-Alexa647 antibody in conjunction with a P815 target cell. Cells were fixed after 45 min of incubation to allow CTLs to form an immunological synapse (IS, indicated by the dashed line) with the target cell (brightfield image, left). Shown are from left to right, two Syb2-mRFP positive CTLs (yellow), one CTL expressing only mFwe2-mTFP (green), the endocytosed anti-RFP-Alexa647 (magenta), and the merged image. Note that upon IS formation only Syb2-mRFP expressing CTLs took up the anti-RFP-Alexa-647 antibody, demonstrating the specificity of the assay. Scale bar 5 µm. **(B)** Western blot analysis of 20 µg lysate of day 5 activated Fwe KO CTL 12-14 h after transfection with the different Flower fusion constructs tagged to mTFP. The blot was probed with anti-DsRed antibody against the mTFP protein. α-Tubulin was used as loading control. **(C)** Fluorescence intensities measured in the CTL's cytoplasm after overexpression of Flower-mTFP fusion constructs in those mouse KO CTLs that were used for endocytosis rescue analysis. Color code of the scatter dot blots correspond to the corresponding line graphs shown in Fig. 4E. Data given as mean ± SD. (*$p < 0.05$, not significant (ns)). **(D)** Time-lapse live snapshots over 15 min of Syb2-mRFP (yellow) of mTFP-mFwe2 and mFwe2-mTFP constructs (green) transfected into Fwe KO CTLs conjugated to P815 target cells in the presence of anti-RFP-Alexa647 antibody (magenta) in the medium. Scale bar 5 µm. **(E)** Quantitative analysis of redistribution of endocytosed Syb2 by anti-RFP-Alexa647 fluorescence from IS into the cytosol for mTFP-mFwe2 and mFwe2-mTFP constructs as shown in (D) in comparison to WT and Flower KO mouse CTLs. Time zero is

defined as the appearance of the first endocytic signal at the IS. Data given as mean ± SEM; Kruskal-Wallis One-way Analysis of Variance on Ranks followed by multiple comparison (Dunn's) was done against Fwe KO as control (KO n=19, N=4 ; WT n=16 (identical to Fig. 4E), mFwe2-mTFP n=16, mTFP-mFwe2 n=16, N=3; $^*p < 0.05$, $^{**}p < 0.01$, $^{***} p < 0.001$).

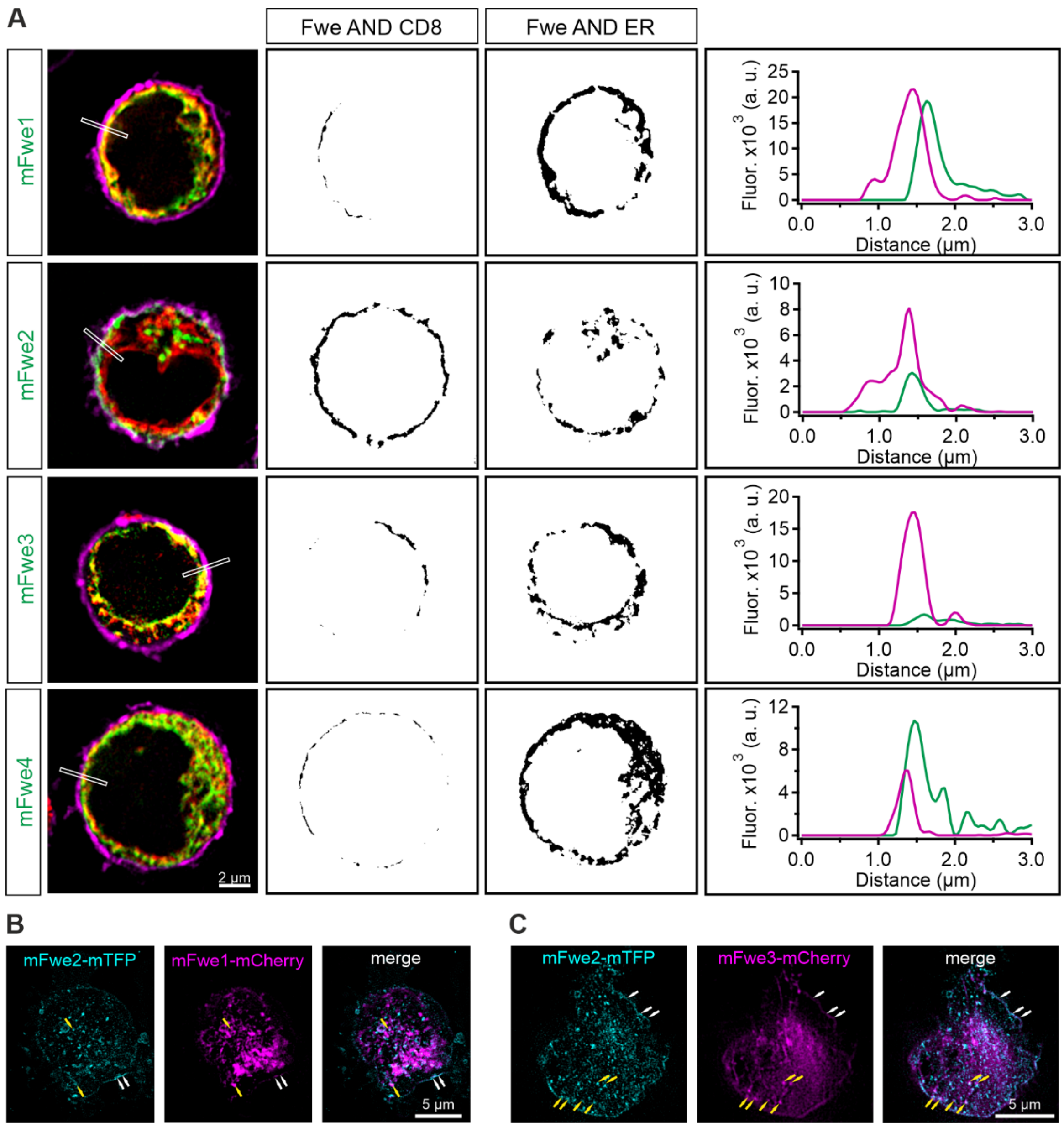

Supplementary Figure 5 (related to Fig. 5): Mouse Flower isoforms polarize to the immunological synapse upon anti-CD3 stimulation. (A) Single plane SIM images of Fwe KO CTLs from Fig. 5 expressing mFwe1-4-mTFP isoforms and ER-mScarletI and stained with the surface marker CD8-Alexa647 are shown in the left panel. Panel two and three show the results of the intersections between masks of Fwe-mTFP and CD8-Alexa647 as well as Fwe-mTFP and ER mScarletI, respectively. These binary images were generated by first thresholding the raw images using the OTSU algorithm in ImageJ to create mask images of each channel. Their intersection was determined with the image calculation function AND. The right panel shows the corresponding line plot of the region marked with open line in the SIM images on the left. The line plots (CD8-Alexa647 (magenta line), Fwe isoforms (green line)) were measured from the outside of the cell to the cytosol. Scale bar 2 µm. **(B, C)** mFwe isoforms colocalize on vesicular structures. SIM images of (**B**) Fwe KO CTLs co-expressing mFwe2-mTFP and mFwe1-mCherry, or (**C**) mFwe2-mTFP and mFwe3-mCherry, on anti-CD3ε-coated coverslips. Cells were fixed after 30 minutes to allow Flower isoforms to polarize to the synapse. The expressed Flower isoforms are observed at the synaptic membrane (white arrows) and colocalize on vesicular structure (yellow arrows).

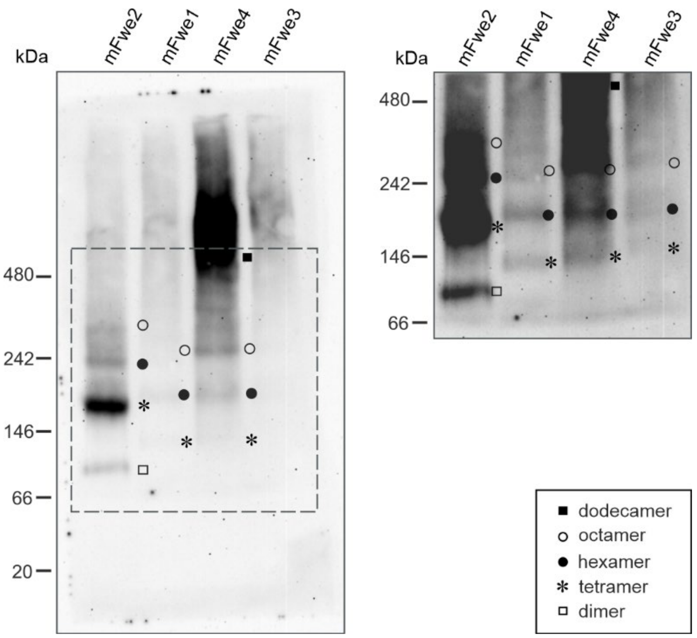

Supplementary Figure 6 (related to Fig. 5): Mouse Flower 2 protein appears primarily as a tetramer or dimer in CTLs after 1 h bead stimulation while the non-rescuers mFwe4 exhibit higher order oligomerization. Fwe KO CTLs of day 5 were transfected with the corresponding mouse Flower fusion constructs tagged with mTFP. After 12-14 h cells were stimulated with fresh beads for 1 h and lysed in a buffer with 1% Triton-X-100 as detergent (see materials and methods). Lysates (10 µg for mFwe-mTFP constructs) were analyzed by blue native polyacrylamide gel electrophoresis (BN-PAGE), western blotting and probed with anti-DsRed antibody to detect mTFP. A representative western blot of BN-PAGE is shown. The inset on the right displays the area extracted from left plot with longer exposure time to better visualize mFwe1 and 3. Different states of oligomerization were marked: dimer (open square), tetramer (star), hexamer (black circle), octamer (open circle), dodecamer (black square). (N=2)

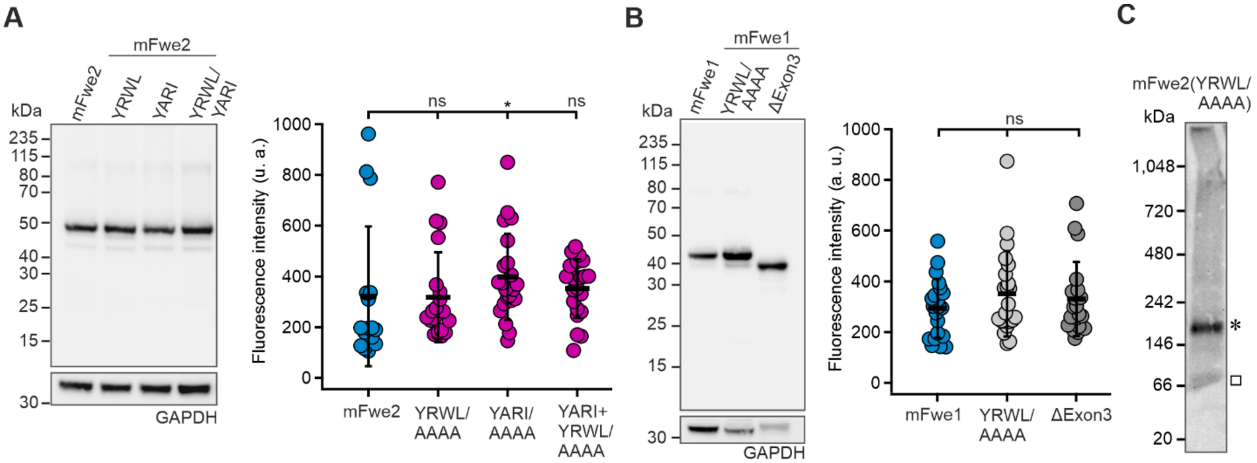

Supplementary Figure 7 (related to Fig. 6): Protein expression of mFwe mutant fusion constructs and their fluorescence intensities in mouse KO CTL are similar. (A-B) Left: Western blot analysis of 20 µg lysate of day 5 activated Fwe KO CTL 12-14 h after transfection with the different Flower fusion constructs tagged to mTFP. The blots were probed with anti-DsRed antibody against the mTFP protein. GAPDH was used as loading control. **(A-B)** Right: mTFP-Fwe construct expression levels in the cells analyzed in the endocytosis rescue experiments. Shown is the mTFP fluorescence intensities in the mFwe KO CTL's cytoplasm after overexpression of the various mFwe-mTFP constructs. Color code of the scatter dot blots correspond to the corresponding line graphs shown in Fig. 6B and Fig. S10C. Data given as mean ± SD. (*p < 0.05, not significant (ns)). **(C)** Oligomerisation states of the mutant mFwe2(YRWL/AAAA) is displayed on a representative western blot of BN-PAGE. Experimental procedure was the same as in Fig. S6. Different states of oligomerization were marked in each western blot as follows: dimers are open square, tetramer are marked by a star. (N=2)

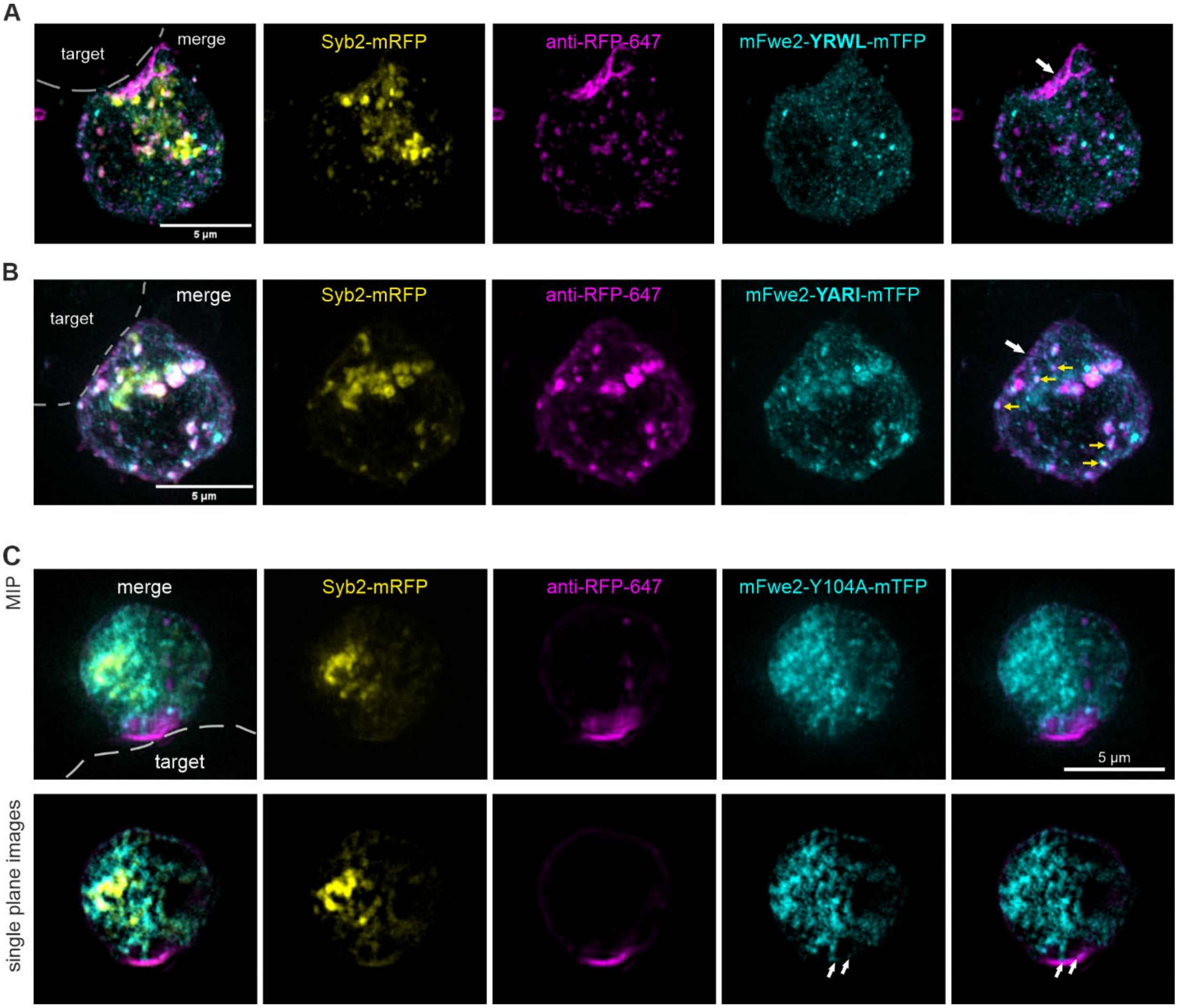

Supplementary Figure 8 (related to Fig. 6, 8): Flower mutants impair the endocytosis of cytotoxic granules. SIM images of Fwe KO CTLs expressing Syb2-mRFP and the indicated Flower mutant constructs. Transfected CTLs were co-incubated with P815 cells in the presence of anti-RFP-647 antibody for 30 minutes. Cells were then fixed and prepared for SIM imaging. **(A)** Maximum intensity projection (MIP) images of CTLs expressing the mFwe2-YRWL-mTFP mutant are shown. The white arrow points to the accumulation of anti-RFP at the immunological synapse (IS) **(B)** MIP images of a CTL expressing the mFwe2-YARI-mTFP mutant are presented. Yellow arrows indicate vesicular structures in which anti-RFP and mFwe2-YARI traffic together, while the white arrows point to the slight accumulation of anti-RFP at the IS. **(C)** MIP and single-plane images of a CTL expressing the mFwe2-Y104A-mTFP mutant are presented. White arrows point to the Y104A mutant, which co-localized with endocytic Syb2 at the IS membrane. T cell–target cell contact sites are marked with stippled lines. Scale bar: 5 µm.

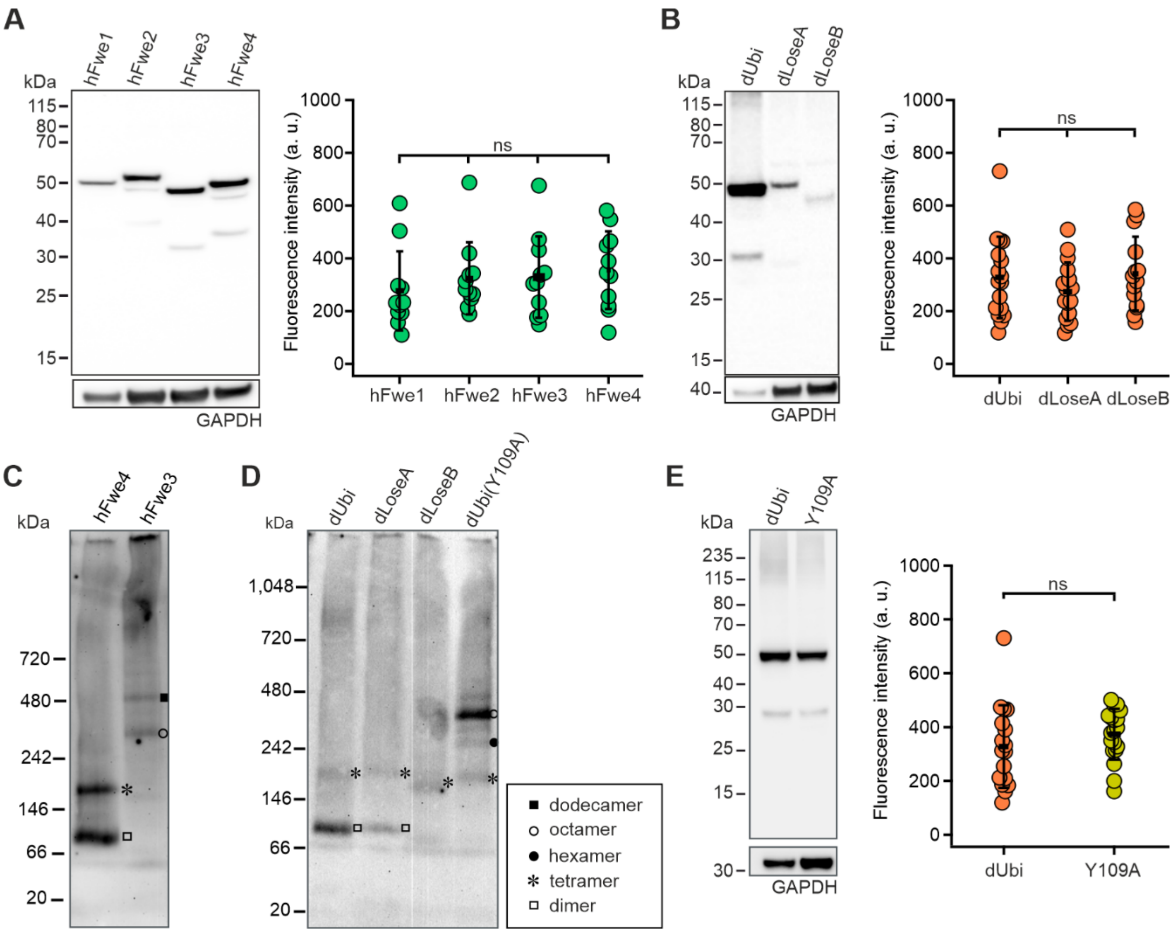

Supplementary Figure 9 (related to Fig. 7): Protein expression of human and *Drosophila* Flower fusion constructs and their fluorescence intensities in mouse KO CTLs are similar. (A, B, E) Left: Western blot analysis of 20 µg lysate of day 5 activated Fwe KO CTLs 12-14 h after transfection with the indicated Flower fusion constructs tagged to mTFP. The blots were probed with anti-DsRed antibody against the mTFP protein. GAPDH was used as loading control. Note that for dFwe constructs (B left) transfection efficiency was low leading to weak band intensity. **(A, B, E) Right**: Fluorescence intensities measured in the CTL's cytoplasm after overexpression of Flower mTFP fusion constructs in those mouse KO CTL used for the endocytosis rescue analysis. Color code of the scatter dot blots correspond to the corresponding line graphs shown in Fig. 7D, F and Fig. 8C. Data given as mean ± SD. (*p < 0.05, not significant (ns)). **(C-D)** Analysis of the oligomerization state of human (C) and *Drosophila* (D) Flower constructs by western blots of BN-PAGE. Experimental procedure was the same as in Fig. S6. Different states of oligomerization were marked in each western blot as shown in E. (N=2)

A

```
Fwe2        MSGSGAAGAAAGPAPPAQEEGMTWWYRWLCRLAGVLGAVSCAISGLFNCVTIHPLNIAAG 60
Fwe1        MSGSGAAGAAAGPAPPAQEEGMTWWYRWLCRLAGVLGAVSCAISGLFNCVTIHPLNIAAG 60
Fwe1(Δex3)  MSGSGAAGAAAGPAPPAQEEGMTWWYRWLCRLAGVLGAVSCAISGLFNCVTIHPLNIAAG 60
            ************************************************************

Fwe2        VWMIMNAFILLLCEAPFCCQFVEFANTVAEKVDRLRSWQKAVFYCGMAIVPIVMSLTLTT 120
Fwe1        VWMIMNAFILLLCEAPFCCQFVEFANTVAEKVDRLRSWQKAVFYCG-------------- 106
Fwe1(Δex3)  VWMI-------------------------------------------------------- 64
            ****

Fwe2        LLGNAIAFATGVLYGLSALGKKGDAISYARIQQQRQQADEEKLAETFEGEL 171
Fwe1        --------------------------------------------------- 106
Fwe1(Δex3)  --------------------------------------------------- 64
```

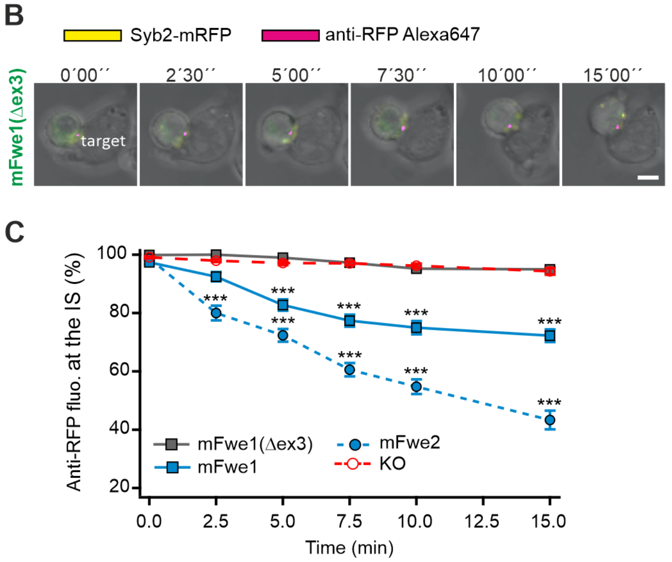

Supplementary Figure 10 (related to Fig. 7)**: Exon 3 is critical for mFwe1 function. (A)** Sequence alignment of mouse Flower isoforms 2, 1, and mFwe1(Δex3) using Clustal Omega. * Indicates conserved amino acids. Amino acids of exon3 are highlighted in yellow. **(B)** Time-lapse live snapshots over 15 min of Syb2-mRFP (yellow) of mFwe1-ΔExon3-mTFP construct (green) transfected into Fwe KO CTL conjugated to P815 target cells in the presence of anti-RFP-Alexa647 antibody (magenta) in the medium. Scale bar: 5 µm. **(C)** Displayed is the quantitative analysis of redistribution of endocytosed Syb2 (anti-RFP-Alexa647) from the IS into the cytosol for mFwe1(Δex3) in comparison to mFwe2-mTFP, mFwe1-mTFP and Flower KO mouse CTLs. Time zero is defined as the appearance of the first endocytic signal at the IS. Data given as mean ± SEM; Kruskal-Wallis One-way Analysis of Variance on Ranks followed by multiple comparison (Dunn's) was done against Fwe KO as control. (KO n=19, N=4 (identical to Fig. 4E); mFwe2 n=16, mFwe1 n=23, mFwe1(Δex3) n=20, N=3), *$p < 0.05$, **$p < 0.01$, *** $p < 0.001$.

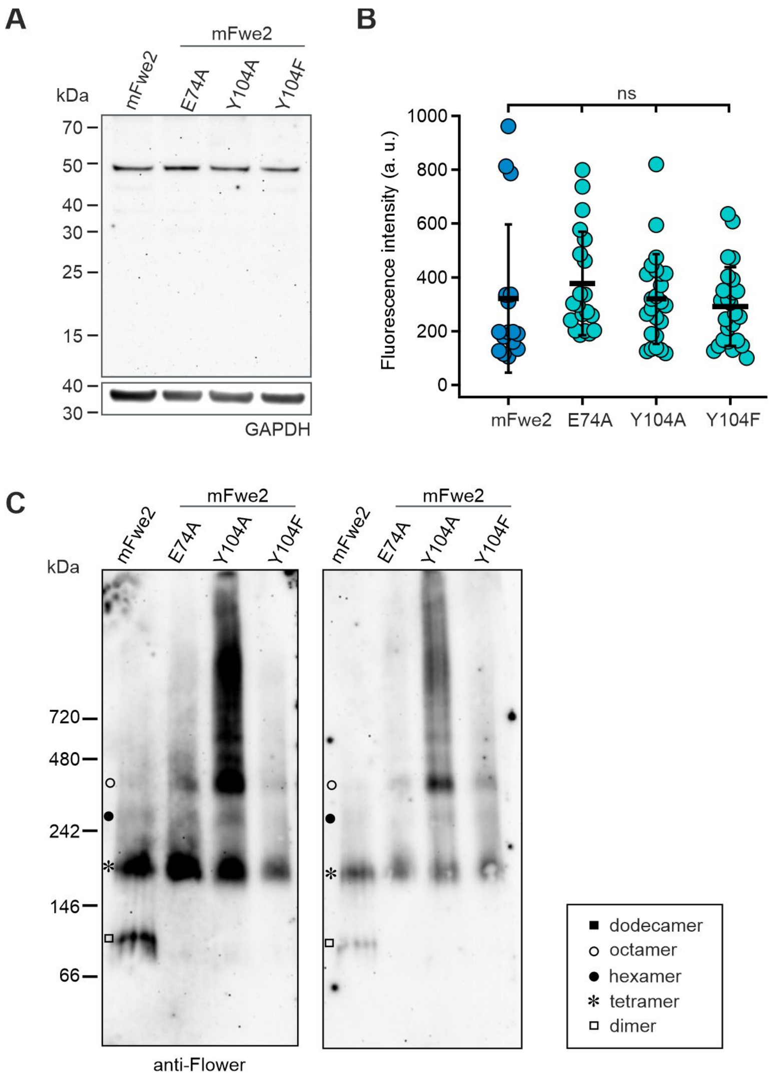

Supplementary Figure 11 (related to Fig. 8): Mutation of the amino acid Y104 of mFwe2 results in higher oligomerization states. (A) Western blot analysis of 20 µg lysate of day 5 activated Fwe KO CTLs 12-14 h after transfection with the different Flower fusion constructs tagged to mTFP. The blots were probed with anti-DsRed antibody against the mTFP protein. GAPDH was used as loading control. **(B)** Fluorescence intensities measured in the CTL's cytoplasm after overexpression of Flower mTFP fusion constructs in mouse KO CTL used for endocytosis rescue analysis. Color code of the scatter dot blots correspond to the corresponding line graphs shown in Fig. 8C. Data given as mean ± SD. (*$p < 0.05$, not significant (ns)). **(C)** Analysis of the oligomerization state of mFwe2 mutant constructs by western blots of BN-PAGE. Experimental procedure was the same as in Fig. S6 except that 7 µl lysate were used. In addition, the blot was not only probed with anti-DsRed antibody (right) but also with anti-Flower antibody (left). Different states of oligomerization were marked in each western blot. (N=2)
